# Modulation of phospho-proteins by interferon-alpha and valproic acid in acute myeloid leukemia

**DOI:** 10.1007/s00432-019-02931-1

**Published:** 2019-05-20

**Authors:** Rakel Brendsdal Forthun, Monica Hellesøy, André Sulen, Reidun Kristin Kopperud, Gry Sjøholt, Øystein Bruserud, Emmet McCormack, Bjørn Tore Gjertsen

**Affiliations:** 1 Centre for Cancer Biomarkers (CCBIO), Department of Clinical Science, Precision Oncology Research Group, University of Bergen, P.O Box 7804, 5020 Bergen, Norway; 2 Department of Internal Medicine, Hematology Section, Haukeland University Hospital, Bergen, Norway; 3 Department of Biomedical Laboratory Sciences and Chemical Engineering, Bergen University College, Bergen, Norway; 4 Department of Clinical Science, Faculty of Medicine and Dentistry, University of Bergen, Bergen, Norway

**Keywords:** AML, IFNα, VPA, Phospho-flow, CyTOF, Phosphoproteome

## Abstract

**Purpose:**

Valproic acid (VPA) is suggested to be therapeutically beneficial in combination with interferon-alpha (IFNα) in various cancers. Therefore, we examined IFNα and VPA alone and in combinations in selected AML models, examining immune regulators and intracellular signaling mechanisms involved in phospho-proteomics.

**Methods:**

The anti-leukemic effects of IFNα and VPA were examined in vitro and in vivo. We mapped the in vitro phosphoprotein modulation by IFNα-2b and human IFNα-Le in MOLM-13 cells by IMAC/2D DIGE/MS analysis and phospho-flow cytometry, and in primary healthy and AML patient-derived PBMCs by CyTOF. In vivo, IFNα-Le and VPA efficacy were investigated in the immunodeficient NOD/Scid IL2γ−/− MOLM-13^Luc+^ mouse model and the syngeneic immunocompetent BNML rat model.

**Results:**

IFNα-2b and IFNα-Le differed in the modulation of phospho-proteins involved in protein folding, cell stress, cell death and p-STAT6 Y641, whereas VPA and IFNα-Le shared signaling pathways involving phosphorylation of Akt (T308), ERK1/2 (T202/T204), p38 (T180/Y182), and p53 (S15). Both IFNα compounds induced apoptosis synergistically with VPA in vitro. However, in vivo, VPA monotherapy increased survival, but no benefit was observed by IFNα-Le treatment. CyTOF analysis of primary human PBMCs indicated that lack of immune-cell activation could be a reason for the absence of response to IFNα in the animal models investigated.

**Conclusions:**

IFNα-2b and IFNα-Le showed potent and synergistic anti-leukemic effects with VPA in vitro but not in leukemic mouse and rat models in vivo. The absence of IFNα immune activation in lymphocyte subsets may potentially explain the limited in vivo anti-leukemic effect of IFNα-monotherapy in AML.

**Electronic supplementary material:**

The online version of this article (10.1007/s00432-019-02931-1) contains supplementary material, which is available to authorized users.

## Background

Acute myeloid leukemia (AML) is a heterogeneous aggressive blood cancer characterized by a block in differentiation, elevated threshold for undergoing apoptosis and excessive proliferation of myeloid progenitor cells (Dohner et al. 2017). Median age of diagnosis is approximately 70 years (Juliusson et al. 2009), and 5 year overall survival is only 5% for patients older than 65 years (Visser et al. 2012), underscoring the need of more effective therapy with acceptable toxicity. IFNα has been tested in AML as induction therapy (Berneman et al. 2010), as a post-remission strategy to prevent recurrence after chemotherapy (Goldstone et al. 2001), in consolidation with allogeneic hematopoietic stem cell transplantation (HSCT) (Klingemann et al. 1991), and as a salvage therapy for patients relapsing upon allogeneic HSCT (Arellano et al. 2007). Case reports showing complete remission after IFNα monotherapy in secondary AML following essential thrombocytosis and/or myelofibrosis may indicate that subsets of patients are particularly sensitive to IFNα (Berneman et al. 2010; Dagorne et al. 2013). IFNα also seems to be effective to prevent relapse in minimal residual disease (MRD) positive patients after HSCT (Mo et al. 2015), while no effect has been reported in children’s relapsed/refractory leukemia (Ochs et al. 1986).

Several formulations of therapeutic IFNα have been available for clinical use. In addition to the most used recombinant IFNα-2b, a human purified preparation of IFNα consisting of six different subtypes (IFNα-Le) has been shown beneficial in melanoma (Stadler et al. 2006). IFNα-Le consists of IFNα1, -α2, -α8, -α10, -α14 and -α21, whereof IFNα2 and IFNα14 are glycosylated. Intriguingly, the IFNα-induced molecular phospho-signaling response has not systematically been characterized in cancer cells, and the anti-leukemic effect of IFNα-Le has previously never been compared with recombinant IFNα-2b.

The combination of IFNα with the histone deacetylase inhibitor valproic acid (VPA) has been reported to be synergistic in several solid cancer models (Jones et al. 2009; Iwahashi et al. 2011; Hudak et al. 2012), suggesting that this combination could represent a valuable novel therapeutic strategy in AML. VPA is an anticonvulsant also used in bipolar disease with well-characterized side effects. Its anti-leukemic effect has been examined in combination with all-*trans* retinoic acid (ATRA) (Trus et al. 2005), 5-azacytidine or low dose cytarabine with responses in up to 20% of the AML patients (Kuendgen et al. 2006; Raffoux et al. 2010; Corsetti et al. 2011; Fredly et al. 2013).

In this study, we compared recombinant and purified human IFNα formulations and found specific regulation of signaling pathways. The combination of IFNα with VPA was synergistic in vitro, but even though in vivo experiments supported the anti-leukemic effect of VPA, we did not find a beneficial effect of IFNα or the combination of IFNα and VPA in vivo.

## Materials and methods

### Cell culture

MOLM-13 (DSMZ, Braunschweig, Germany) and IPC-81 cells [obtained from Dr. Michel Lanotte (Lacaze et al. 1983)] were incubated with; 250 or 2000 IU/mL IFNα-2b (Intron A, Schering-Plough, Kenilworth, New Jersey, USA), 250 or 2000 IU/mL IFNα-Le (Multiferon, generously provided by Sobi Swedish Orphan Biovitrum, Stockholm, Sweden), 1 mM VPA (Desitin Pharma AS, Hamburg, Germany) or a combination of 2000 IU/mL IFNα-2b or IFNα-Le and 1 mM VPA for 15 min or 48 h. AML patient peripheral blood mononuclear cells (PBMCs, *n* = 12; six normal karyotype, six complex karyotype, Table 1) and healthy donor PBMCs (*n* = 5) were collected after written informed consent in compliance with the Declaration of Helsinki (REK2016/253, REK2012/2247). PBMCs were isolated by Ficoll separation (Sigma-Aldrich, Darmstadt, Germany) and cryopreserved in liquid nitrogen for long-term storage. The cells were thawed, centrifuged for 5 min at 300*g* before incubation for 15 min in StemSpan (STEMCELL Technologies, Inc. Vancouver, Canada) added 9% DMEM (Sigma-Aldrich) and 1% DNase I Solution (STEMCELL Technologies). Cells were then plated at 1x10^6^ cells/mL and added media, 2000 IU/mL IFNα-2b, 1 mM VPA or a combination of IFNα-2b and VPA for 48 h before counting, washing with Maxpar PBS (Fluidigm, San Francisco, CA, USA), fixed with 2% paraformaldehyde (PFA) in Maxpar PBS for 10 min at 37 °C, followed by freezing at − 80 °C for storage prior to analysis.

**Table 1:** Donor cell characteristics

Sample information					Cell populations in non-treated sample (% of total)										
ID	Group	Karyotype	FLT3	NPM1	Blasts	B cells	Mono-cytes	pDCs	NK cells	NKT cells	DNT cells MC15	DNT cells MC3	CD4^+^CD7^−^ T cells	CD4^+^ T cells	CD8^+^ T cells
P1	AML patient	Complex^a^	Wt	Wt	6.25	1.45	52.61	0	7.98	10.38	0	0	4.34	16.98	0
P2	AML patient	Complex	ITD	Ins	52.42	0.43	8.56	0.21	3.01	16.87	0	0	2.21	10.52	0
P3	AML patient	Complex	Wt	Wt	34.22	0.62	4.70	0	0	6.28	0	22.19	18.64	0	13.35
P4	AML patient	Complex	Wt	Wt	96.89	0	0	0.65	0	0	0	0	0	0	2.45
P5	AML patient	Complex	Wt	Ins	82.10	4.99	0	0.22	0	0	0	9.19	0.08	2.05	1.37
P6	AML patient	Complex	Wt	Wt	90.85	0.70	0	0.37	0	0	0	5.69	0	0	2.39
P7	AML patient	Normal	Wt	Wt	82.22	0	0	0	0	0	0	14.84	0	0	2.94
P8	AML patient	Normal	ITD	Ins	90.36	0	0	0.50	0	0	0	6.59	0	0	2.55
P9	AML patient	Normal	ITD	Ins	86.33	2.80	0	0.25	0	0	0	7.18	0.20	0	3.24
P10	AML patient	Normal	ITD	Ins	95.46	0	0	0	0	0	0	4.54	0	0	0
P11	AML patient	Normal	ITD	Wt	95.64	0	1.09	0	0	0	0	3.27	0	0	0
P12	AML patient	Normal	ITD	Ins	97.11	0.59	0	0.24	0	0	0	1.32	0	0	0.74
D1	Healthy donor	Normal	Wt	Wt	–	6.27	5.18	0.08	22.2	16.24	2.5	12.77	3.42	24.42	6.43
D2	Healthy donor	Normal	Wt	Wt	–	7.64	3.49	0.31	19.27	13.05	8.16	6.74	5.50	16.40	18.23
D3	Healthy donor	Normal	Wt	Wt	–	9	1.64	0.23	9.69	15.40	3.47	22.25	3.47	29.50	5.18
D4	Healthy donor	Normal	Wt	Wt	–	9.07	0.63	0	24	19.04	0.42	3.91	4.46	38.46	0
D5	Healthy donor	Normal	Wt	Wt	–	7.27	2.30	0.24	8.62	15.35	6.65	1.34	4.55	52.36	0

^a^Defined as complex based on findings in (Breems et al. 2008)

### Staining of primary cells for mass cytometry (CyTOF)

To assure comparability across samples from different donors, the samples were barcoded using a commercially available metal barcoding kit (Fluidigm), according to the manufacturer’s instructions. Twenty samples were multiplexed, making a total of four pooled samples, each containing AML patient and healthy donor samples.

The samples were subsequently stained with the antibody panels (Online Resource Tables 1 and 2) following the MaxPar phospho-protein staining protocol (Fluidigm), with minor adjustments. Briefly, amendments to the protocol include; Fc receptors blocking was done using Human IgG (Octagam^®^, Octapharma, Lachen, Switzerland) for 20 min at RT. To block nonspecific antibody binding to eosinophils (Rahman et al. 2016), samples were pre-incubated with 100 IU heparin sodium (Wockhardt, Wrexham, UK) for 20 min, and subsequently stained with antibody cocktails (Online Resource Tables 1 and 2) in the presence of 100iU heparin. DNA intercalation stain (iridium, Fluidigm) was diluted at 1:1250 in 4% PFA in Maxpar PBS, and samples were incubated at 4 °C overnight. After staining, samples were resuspended in a 1:8 solution of Maxpar cell acquisition solution (Fluidigm) and EQ™ four element calibration beads (Fluidigm).

Acquisition of samples was done using a Helios mass cytometer (Fluidigm). After acquisition, the collected data was normalized to EQ bead standard (Finck et al. 2013) and exported to FCS3 files. Data was subsequently uploaded to Cytobank Cellmass software (Cytobank Inc, Santa Clara, CA, USA) and evaluated using established methods (Diggins et al. 2015; Levine et al. 2015). Phenograph was run with the Cyt interphase in Matlab (Mathworks, Natick, MA, USA), see Online Resource Fig. 1 for gating strategy. The non-parametric Kruskal–Wallis *H* test was used to determine statistical significance (*p *< 0.05) using R software (R Core Team (2017), R Foundation for Statistical Computing, Vienna, Austria).

### Phospho-flow cytometry staining and analysis

Treated MOLM-13 cells were washed in 0.9% NaCl, fixated in 1.6% PFA for 15 min at room temperature (RT), added ice-cold methanol and stored at − 80 °C prior to analysis. Samples were fluorescently barcoded using Pacific Blue and Pacific Orange (Molecular Probe, Eugene, OR, USA), as described previously (Krutzik and Nolan 2006). The Student’s unpaired, two-tailed t test (GraphPad, GraphPad Software, Inc., La Jolla, CA, USA) was used to determine statistical significance (*p *< 0.05). Primary antibodies are described in Online Resource Table 3.

### Viability and cell death assays

Viability was determined using Annexin-V Alexa Fluor 488 (Life Technologies Ltd, Paisley, UK) and Propidium Iodide (PI) (Sigma-Aldrich), and cell death was analyzed by Hoechst 33342 DNA staining (Calbiochem, Merck KGaA, Darmstadt, Germany) as described in the Online Resources.

### IMAC phosphoprotein purification, two-dimensional differential gel electrophoresis, gel analysis and protein identification by mass spectrometry (IMAC/2D DIGE/MS)

Phosphoproteins were enriched using the PhosphoProtein Purification Kit (Qiagen, Hilden, Germany) as recommended by the manufacturer. In short, 1 × 10^7^ cells were lysed after IFNα treatment (48 h) as previously described (Forthun et al. 2012). Subsequently, phosphoprotein samples were covalently labeled with fluorescent CyDyes (GE Healthcare, Chicago, Illinois, US) in a minimal labeling reaction (400 pmol dye:50 µg protein) and isoelectrically focused on pH 3–11 DryStrip Immobiline gel strips (GE Healthcare) prior to second dimension gel electrophoresis and mass spectrometry identification as described in the Online Resources.

### Animals

Fifteen 240–320 g male Brown Norwegian rats (BN/mcwi) (Charles River Laboratories, Wilmington, MA, USA) and 40 20–25 g female NOD/Scid IL2 γ−/− (NSG) mice (the Vivarium, University of Bergen, Norway, originally a generous gift from Prof. Leonard D. Shultz, Jackson Laboratories, Bar Harbour, Maine, USA) were injected intravenously in the lateral tail vein with 10 million BNML cells or 5 million MOLM-13^Luc+^ cells, respectively. Animals were dosed with VPA intraperitoneally (BNML; 400 mg/kg, *n* = 4, MOLM-13^Luc+^; 350 mg/kg, *n* = 7), IFNα-Le by subcutaneous injections (BNML; 0.8x10^6^ IU/kg—human equivalent dose 0.13 × 10^6^ IU/kg, *n* = 4, MOLM-13^Luc+^; 1 × 10^6^ IU/kg—human equivalent dose 0.08 × 10^6^ IU/kg, *n* = 7), or a combination of VPA and IFNα-Le (BNML; *n* = 4, MOLM-13^Luc+^; *n* = 7). Control groups (BNML; *n* = 3, MOLM-13^Luc+^; *n* = 7) received subcutaneous injections of 0.9% NaCl (Fresenius Kabi AG, Bad Homburg, Germany). Calculation of IFNα-Le doses was based on relevant therapeutic doses and is described in the Online Resources. Treatment was initiated day 10 (BNML) or day 7 (MOLM-13^Luc+^), with VPA 5 days successively per week, and IFNα-Le three times a week (day 1, 3 and 5) for a total of 4 weeks. MOLM-13^Luc+^ mice were imaged by bioluminescent optical imaging once a week as described in the Online Resources. Animals were sacrificed at humane endpoint, defined as loss of body weight (mice 10%, rats 15%), ataxia, paralysis of hind or fore limbs, lethargy or dehydration. Survival ratios were investigated by Log-rank (Mantel-Cox) Test on Kaplan–Meier curves (GraphPad). All applicable international, national and institutional guidelines for the care and use of animals were followed for all animal studies. The animal experiments were reviewed and approved by The Norwegian Animal Research Authority under study permit number 2009 1955 and 2015 7229 and conducted according to The European Convention for the Protection of Vertebrates Used for Scientific Purposes.

## Results

### Phosphoproteome analysis of IFNα-Le and IFNα-2b

**Fig. 1:**
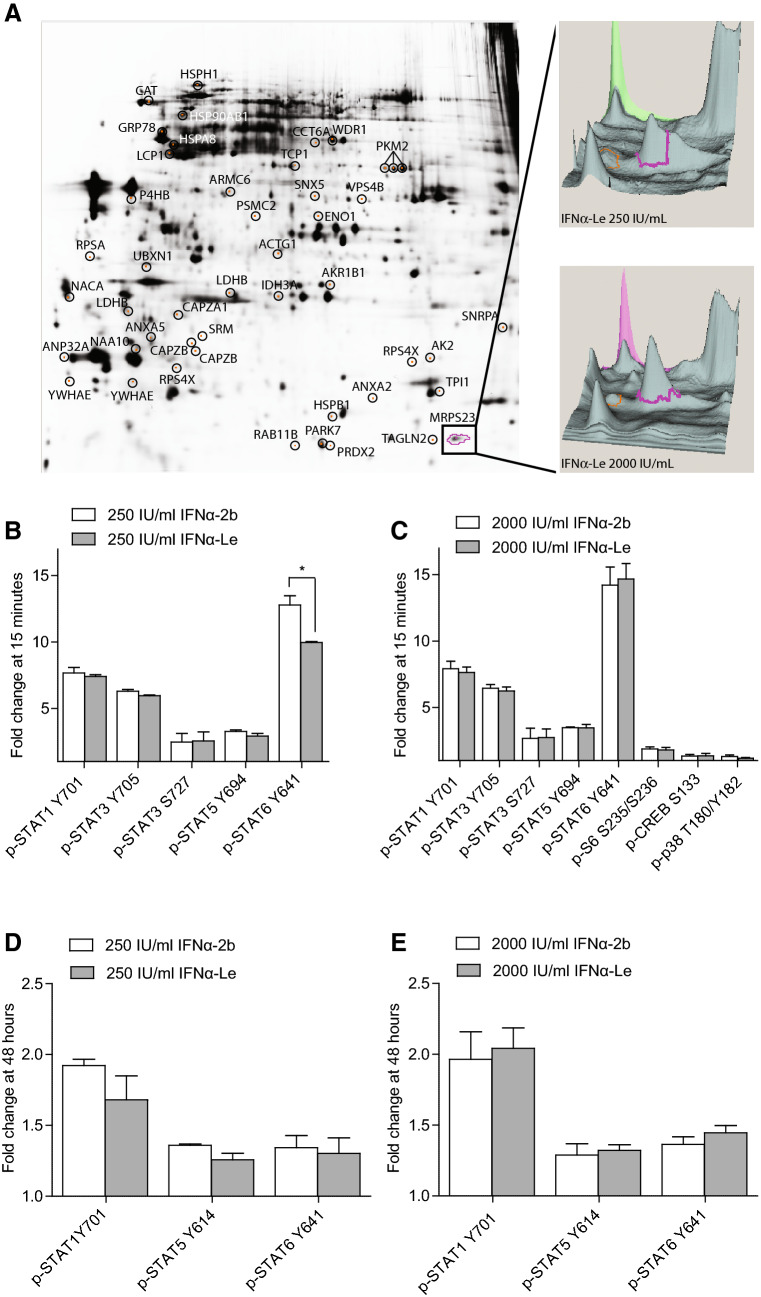
Phospho-signaling induced by recombinant IFNα-2b and human IFNα-Le in human AML MOLM-13 cells. **a** MOLM-13 cells were treated with 200 or 2000 IU/mL IFNα-2b or IFNα-Le for 48 h. Forty-seven proteins were significantly differentially regulated by IFNα and are encircled and identified by protein name. The highlighted protein MRPS23 is visualized by the three-dimensional visualization of cyanine-labeled protein emission intensities indicating the abundance of MRPS23. MOLM-13 cells were further analyzed by flow cytometry after treatment with **b** 250 IU/mL or **c** 2000 IU/mL IFNα-2b or IFNα-Le (*n* = 3) for 15 min, and with **d** 250 IU/mL or **e** 2000 IU/mL IFNα-2b or IFNα-Le for 48 h (*n* = 3). Only proteins significantly differently expressed from the control samples (*p * ≤ 0.05), with a minimum fold change of 1.3 are displayed

We investigated the difference in phosphoprotein regulation between the two IFNα compounds IFNα-Le and IFNα-2b by immobilized affinity chromatography (IMAC) and 2D DIGE in the human AML cell line MOLM-13 (48 h treatment). 2D DIGE showed a total of 47 proteins with higher than 1.3 fold change and a significance level of *p *≤0.05 between the compounds (Fig. 1a, Tables 2 and 3). Only nascent polypeptide-associated complex subunit alpha (NACA) and 40S ribosomal protein SA (RPSA) were modulated at both 250 and 2000 IU/mL IFNα-2b. For IFNα-Le only F-actin-capping protein subunit beta (CAPZB) and actin cytoplasmic 2 (ACTG1) were modulated at 250 and 2000 IU/mL (Table 2). The majority of the IFNα regulated proteins demonstrated a down-regulation after low dose treatment. IFNα-2b at 250 IU/mL induced down-regulation of proteins involved in cell cycle [Ras-related protein Rab-11B (RAB11B)], DNA damage response [26S protease regulatory subunit 7 (PSMC2)] and immune response [F-actin-capping protein subunit alpha-1 (CAPZA1)]. At 2000 IU/mL, up-regulation of adenylate kinase 2 (AK2), a protein necessary for the hematopoiesis (Pannicke et al. 2009) and unfolded protein response (UPR) (Burkart et al. 2011), was found. This was accompanied by down-regulation of proteins involved in transcription and translation [NACA and 40S ribosomal protein SA (RPSA)], as well as oxidative stress response protein peroxiredoxin-2 (PRDX2). IFNα-Le regulated the expression of proteins involved in protein folding, stress response and programmed cell death even at 250 IU/mL (T-complex protein subunit zeta (CCT6A), aldose reductase (AKR1B1) and pyruvate kinase isozymes M1/M2 (PKM2), respectively). Additionally, proteins involved in energy production [(l-lactate dehydrogenase B chain (LDHB) and isocitrate dehydrogenase (NAD) subunit alpha (IDH3A)] were down-regulated. At 2000 IU/mL only cytoskeletal protein ACTG1 was down-regulated, whilst adapter protein 14-3-3 protein epsilon (YWHAE), actin regulator CAPZB, UPR-response Heat shock protein 105 kDa (HSPH1) and gene expression regulator acidic leucine-rich nuclear phosphoprotein 32 family member A (ANP32A) were up-regulated.

**Table 2:** Differently expressed proteins in control versus IFNα treated MOLM-13 cells

No.	Protein	UniProtKB ID	DeCyder number	*p* value	Fold change	Biological process
*250* *IU/mL IFNα-2b*						
1	14-3-3 protein epsilon (YWHAE)	P62258	2864	0.016	1.45	Cell cycle transition, apoptosis, membrane organization
2	Armadillo repeat-containing protein 6 (ARMC6)	Q6NXE6	1602	0.017	− 1.32	Unknown
3	Nascent polypeptide-associated complex subunit alpha (NACA)	Q13765	2377	0.02	− 1.44	DNA dependent transcription, translation
4	Vacuolar protein sorting-associated protein 4B (VPS4B)	O75351	1659	0.023	− 1.30	Cell cycle, ATP catabolic process
5	40S ribosomal protein S4, X isoform (RPS4X)	P62701	2772	0.025	1.44	Translation, positive regulation of proliferation
6	UBX domain-containing protein 1 (UBXN1)	Q04323	2165	0.026	1.60	Negative regulation of protein ubiquitination
7	Triosephosphate isomerase (TPI1)	P60174	2942	0.029	1.40	Glycolysis, metabolic process
8	Ras-related protein Rab-11B (RAB11B)	Q15907	3213	0.032	− 1.58	GTPase mediated signal transduction, cell cycle
9	26S protease regulatory subunit 7 (PSMC2)	P35998	1805	0.039	− 1.84	DNA damage response, negative regulation of apoptosis, cell cycle transition
10	F-actin-capping protein subunit alpha-1 (CAPZA1)	P52907	2493	0.047	− 1.42	Actin cytoskeleton organization, blood coagulation, immune response
11	40S ribosomal protein SA (RPSA)	P08865	2087	0.047	− 1.30	Translation, RNA metabolic process
*2000* *IU/mL IFNα-2b*						
1	Nascent polypeptide-associated complex subunit alpha (NACA)	Q13765	2377	0.011	− 1.31	DNA dependent transcription, translation
2	Alpha-enolase (ENO1)	P06733	1789	0.017	− 1.31	Glycolysis, negative regulation of cell growth
3	T-complex protein 1 subunit alpha (TCP1)	P17987	1355	0.021	− 1.35	Protein folding, tubulin complex assembly, cellular protein metabolic process
4	Peroxiredoxin-2 (PRDX2)	P32119	3214	0.023	− 1.30	Response to oxidative stress, negative regulation of apoptosis
5	Adenylate kinase 2 (AK2)	P54819	2753	0.047	1.37	ATP metabolic process, oxidative phosphorylation
6	40S ribosomal protein SA (RPSA)	P08865	2087	0.05	− 1.55	Translation, RNA metabolic process
*250* *IU/mL IFNα-Le*						
1	Protein disulfide-isomerase (P4HB)	P07237	1663	0.00028	1.46	Protein folding, lipoprotein metabolic process, cell redox homeostasis
2	T-complex protein subunit zeta (CCT6A)	P40227	1137	0.003	− 1.52	Protein folding, protein transport
3	F-actin-capping protein subunit beta (CAPZB)	P47756	2679	0.0063	1.43	Actin cytoskeleton organization, blood coagulation
4	L-lactate dehydrogenase B chain (LDHB)	P07195	2474	0.0067	− 1.34	Glycolysis, oxidation–reduction process
5	F-actin-capping protein subunit beta (CAPZB)	P47756	2719	0.009	1.41	Actin cytoskeleton organization, blood coagulation
6	U1 small nuclear ribonucleoprotein A (SNRPA)	P09012	2589	0.0099	1.30	Gene expression, nuclear mRNA splicing
7	Aldose reductase (AKR1B1)	P15121	2292	0.011	− 1.31	Response to stress, carbohydrate metabolic process, daunorubicin and doxorubicin metabolic process
8	UBX domain-containing protein 1 (UBXN1)	Q04323	2165	0.015	1.89	Negative regulation of protein ubiquitination
9	F-actin-capping protein subunit alpha-1 (CAPZA1)	P52907	2493	0.016	− 1.56	Glycolysis, oxidation–reduction process
10	Triosephosphate isomerase (TPI1)	P60174	2942	0.016	1.56	Glycolysis, metabolic process
11	Isocitrate dehydrogenase (NAD) subunit alpha (IDH3A)	P50213	2367	0.021	− 1.46	Carbohydrate metabolic process, oxidation–reduction process
12	Actin, cytoplasmic 2 (ACTG1)	P63261	2075	0.022	− 1.60	Immune response, cellular membrane organization
13	L-lactate dehydrogenase B chain (LDHB)	P07195	2331	0.022	− 1.31	Glycolysis, oxidation–reduction process
14	Pyruvate kinase isozymes M1/M2 (PKM2)	P14618	1380	0.026	− 1.41	Response to hypoxia, programmed cell death, ATP biosynthetic process
15	Pyruvate kinase isozymes M1/M2 (PKM2)	P14618	1381	0.028	− 1.43	Response to hypoxia, programmed cell death, ATP biosynthetic process
16	N-alpha-acetyltransferase 10 (NAA10)	P41227	2695	0.028	− 1.46	Protein amino acid acetylation
17	Annexin A2 (ANXA2)	P07355	2977	0.036	− 1.36	Angiogenesis, collagen fibril organization
*2000* *IU/mL IFNα-Le*						
1	14-3-3 protein epsilon (YWHAE)	P62258	2864	0.00047	1.53	Cell cycle transition, apoptosis, membrane organization
2	F-actin-capping protein subunit beta (CAPZB)	P47756	2679	0.0012	1.60	Actin cytoskeleton organization, blood coagulation
3	Heat shock protein 105 kDa (HSPH1)	Q92598	436	0.016	1.34	Unfolded protein response, positive regulation of NK cell activation
4	Acidic leucine-rich nuclear phosphoprotein 32 family member A (ANP32A)	P39687	2739	0.018	1.36	Regulation of DNA dependent gene expression, RNA metabolic process
5	Actin, cytoplasmic 2 (ACTG1)	P63261	2075	0.029	− 1.36	Immune response, cellular membrane organization

Positive fold change indicates higher protein expression in IFNα treated cells compared to control treatment; negative fold change indicates lower protein expression by IFNα treatment compared to control treatment. DeCyder number refers to ID assigned by the DeCyder software; *p* value was obtained by Students *T* test

**Table 3:** Differently expressed proteins in MOLM-13 cells treated with IFNα-Le versus IFNα-2b

No.	Protein	UniProtKB ID	DeCyder number	*p* value	Fold change	Biological process
*250* *IU/mL IFNα-Le versus IFNα-2b*						
1	L-lactate dehydrogenase B chain (LDHB)	P07195	2331	0.00019	1.41	Glycolysis, oxidation–reduction process
2	Aldose reductase (AKR1B1)	P15121	2292	0.00079	1.31	Response to stress, carbohydrate metabolic process
3	N-alpha-acetyltransferase 10 (NAA10)	P41227	2695	0.0039	1.32	Protein amino acid acetylation
4	Pyruvate kinase isozymes M1/M2 (PKM2)	P14618	1376	0.03	1.40	Response to hypoxia, programmed cell death, ATP biosynthetic process
5	Catalase (CAT)	P04040	646	0.038	1.30	Hydrogen peroxide catabolic process, negative regulation of apoptosis, cell division
6	Pyruvate kinase isozymes M1/M2 (PKM2)	P14618	1381	0.04	1.39	Response to hypoxia, programmed cell death, ATP biosynthetic process
7	26S protease regulatory subunit 7 (PSMC2)	P35998	1805	0.05	− 1.76	DNA damage response, negative regulation of apoptosis, cell cycle transition
*2000* *IU/mL IFNα-Le versus IFNα-2b*						
1	14-3-3 protein epsilon (YWHAE)	P62258	2864	0.00014	− 1.47	Cell cycle transition, apoptosis, membrane organization
2	28S ribosomal protein 23 (MRPS23)	Q9Y3D9	3180	0.00032	− 1.30	Translation
3	40S ribosomal protein S4, X isoform (RPS4X)	P62701	2797	0.00093	1.36	Translation, positive regulation of proliferation
4	Plastin-2 (LCP1)	P13796	1244	0.0015	− 1.48	Protein folding, response to stress, cell cycle, ATP catabolic process
5	Sorting nexin-5 (SNX5)	Q9Y5X3	1628	0.0057	− 1.43	Protein transport, cell communication
6	Acidic leucine-rich nuclear phosphoprotein 32 family member A (ANP32A)	P39687	2739	0.0076	− 1.56	Regulation of DNA dependent gene expression, RNA metabolic process
7	Transgelin-2 (TAGLN2)	P37802	3189	0.0077	− 1.43	Actin organization, muscle organ development
8	Heat shock protein HSP 90-beta (HSP90AB1)	P08238	829	0.0097	− 1.51	Protein folding, response to stress, activation of innate immune response, regulation of type I IFN mediated signaling pathway
9	Alpha-enolase (ENO1)	P06733	1789	0.013	− 1.50	Glycolysis, negative regulation of cell growth
10	Protein deglycase DJ-1 (PARK7)	Q99497	3207	0.015	− 1.39	Autophagy, negative regulation of cell death, response to stress, proteolysis
11	14-3-3 protein epsilon (YWHAE)	P62258	2866	0.023	− 1.43	Cell cycle transition, apoptosis, membrane organization
12	Spermidine synthase (SRM)	P19623	2651	0.034	1.33	Polyamine metabolic process, spermidine biosynthetic process
13	Heat shock protein beta-1 (HSPB1)	P04792	3078	0.034	− 1.67	Angiogenesis, anti-apoptosis, response to stress, response to unfolded protein
14	WD repeat-containing protein 1 (WDR1)	O75083	1104	0.035	− 1.31	Platelet degranulation and activation
15	78 kDa glucose-regulated protein (HSPA5)	P11021	3366	0.04	− 1.42	Platelet degranulation and activation, anti-apoptosis, unfolded protein response, negative regulation of TGFβ receptor signaling pathway, positive regulation of protein ubiquitination
16	Heat shock cognate 71 kDa protein (HSPA8)	P11142	1158	0.041	− 1.41	Protein folding, response to stress, response to unfolded protein, cell cycle, ATP catabolic process
17	T-complex protein 1 subunit alpha (TCP1)	P17987	1355	0.043	− 1.44	Protein folding, tubulin complex assembly, cellular protein metabolic process
18	Annexin A5 (ANXA5)	P08758	2652	0.045	− 1.31	Anti-apoptosis, signal transduction

Positive fold change indicates higher protein expression in IFNα-2b treated cells compared to IFNα-Le treatment; negative fold change indicates higher protein expression by IFNα-Le treatment compared to IFNα-2b treatment. DeCyder number refers to ID assigned by the DeCyder software; *p* value was obtained by Students *T* test

The expression differences induced by IFNα-2b and IFNα-Le demonstrated no overlap between proteins regulated at low and high dose (Table 3). At 250 IU/mL, 6 of 7 proteins had lower expression after IFNα-Le treatment, whilst only PSMC2 was regulated by IFNα-2b (Online Resource Table 4). This effect was reversed at 2000 IU/mL where 16 of 18 proteins showed higher expression after IFNα-Le treatment compared to IFNα-2b, exemplified by up-regulation by IFNα-Le for YWHAE and ANP32A, or down-regulation by IFNα-2b for alpha-enolase (ENO1), heat shock protein beta-1 (HSPB1) and T-complex protein 1 subunit alpha (TCP1).

### Altered intracellular signaling by IFNα-Le and IFNα-2b

To investigate proteins known to be regulated by IFNα, we explored early (15 min) and late (48 h) effects on phosphorylation of signaling proteins involved in cell cycle progression and cell death pathways, as well as IFNα-regulated phosphoproteins in the AML cell line MOLM-13 by phospho-flow cytometry (antibody overview in Online Resource Table 3). Only proteins with a fold change ≥ 1.3 (*p *≤ 0.05) compared to untreated control cells were regarded regulated by the drug treatment. After 15 min exposure, both 250 and 2000 IU/mL IFNα induced phosphorylation of STAT1 (pY701), STAT3 (pY705, pS727), STAT5 (pY694) and STAT6 (pY641) (Fig. 1b, c, Online Resource Fig. 2A). In addition, the high dose induced phosphorylation of CREB (pS133), as well as S6 ribosomal protein (S6) (pS235/pS236) and the known IFNα effector MAP kinase p38 (pT180/pY182) (Fig. 1c). All proteins were similarly regulated by the two drugs except for STAT6, which showed significantly higher phosphorylation (*p *= 0.002) by IFNα-2b compared to IFNα-Le (Fig. 1b).

After 48 h, only STAT1, STAT5 and STAT6 showed increased phosphorylation compared to control cells (Fig. 1d, e, Online Resource Fig. 2B). Both IFNα-2b and IFNα-Le resulted in significantly increased phosphorylation of STAT3, p38, ERK1/2, NFκB and p53 (pS15) at the high dose treatments compared to control cells, however, below threshold limits (Online Resource Fig. 2). No differences in protein phosphorylation could be detected between IFNα-2b and IFNα-Le at 48 h.

### IFNα-Le induces cell death more efficiently than recombinant IFNα-2b

**Fig. 2:**
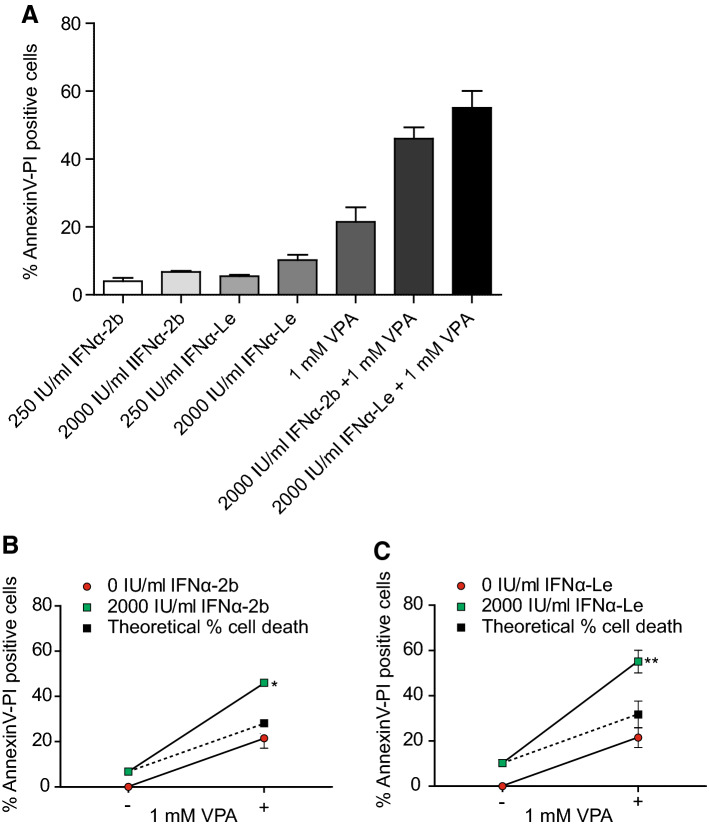
IFNα induce cell death in human MOLM-13 AML cells. Viability was investigated by Annexin-V/PI after 48 h treatment with recombinant IFNα-2b, IFNα-Le and/or 1 mM VPA (*n* = 3). Cell death percentages were normalized to control cells. **a** MOLM-13 cells showed statistically significant increased percent cell death when treated with IFNα or VPA (Student’s unpaired, two tailed *t* test). Combining 1 mM VPA with 2000 IU/ml IFNα resulted in synergism compared to single treatments for both **b** IFNα-2b (two-way ANOVA, *p =0.009) and **c** IFNα-Le (two-way ANOVA, **p = 0.001), as compared to the theoretical additive levels of cell death

Since IFNα-2b and IFNα-Le differed in the regulation of both known and previously unknown IFNα-regulated proteins, we investigated the difference in cell death induction by the two drugs. VPA and IFNα have been reported to act synergistically in several cancer models (Jones et al. 2009; Iwahashi et al. 2011; Hudak et al. 2012), and we, therefore, combined the two drugs with the aim of increasing the modest apoptotic effects of IFNα. MOLM-13 cells were treated for 48 h and analyzed by Hoechst (Online Resource Fig. 3) and Annexin-V/PI staining (Fig. 2). We found that both IFNα-2b and IFNα-Le induced a low but significant increase in apoptosis compared to the control. Whilst increasing the concentration of IFNα-2b from 250 to 2000 IU/mL did not result in significantly increased levels of cell death, 2000 IU/mL IFNα-Le caused elevated levels of cell death (*p *=0.04) (Fig. 2a). Combining 2000 IU/mL IFNα-2b with 1 mM VPA increased the levels of cell death synergistically (46.0%, *p *=0.01) (Fig. 2b), whilst the combination of 2000 IU/ml IFNα-Le with 1 mM VPA was more efficient at inducing cell death (55.1%, *p *= 0.009) (Fig. 2c). For the rat IPC-81 cell line, VPA significantly induced cell death compared to the control (Online Resource Fig. 4A). However, no effect was seen on apoptosis by either IFNα drugs, even though 2000 IU/mL IFNα-Le induced STAT1 (pY701) phosphorylation (Online Resource Fig. 4B).

### Phosphoprotein signaling by the valproic acid/IFNα-Le combination

**Fig. 3:**
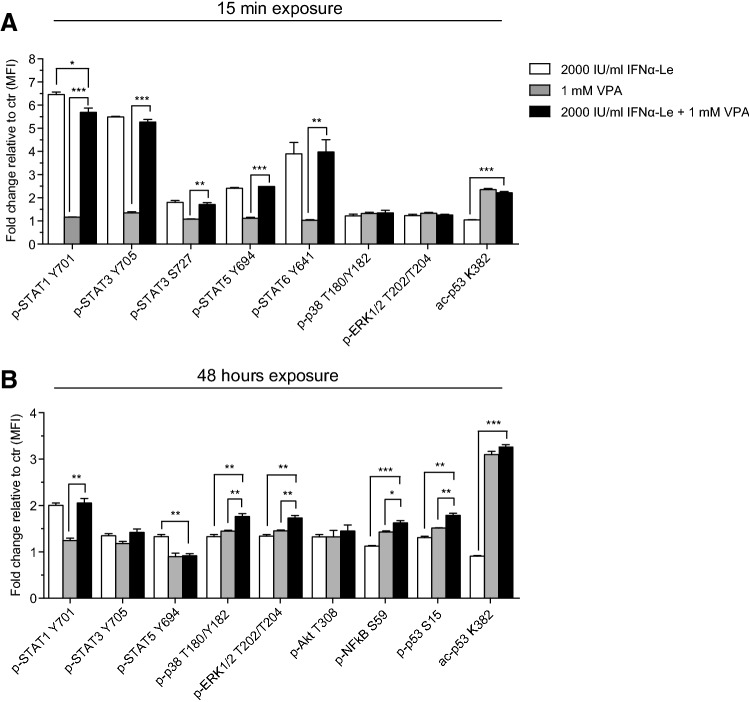
Phospho-signaling induced by VPA and IFNα-Le in MOLM-13 cells. MOLM-13 cells (*n* = 3) were treated with VPA (1 mM) and/or IFNα-Le (2000 IU/mL) and analyzed by flow cytometry. **a** Significantly different expressed proteins after 15 min (*p *≤0.05, fold change ≥ 1.3). **b** Significantly different expressed proteins after 48 h (*p *≤0.05, fold change ≥ 1.3). Scale describes fold change log_2_ compared to untreated control cells (Students unpaired two-tailed *t* test, *p <0.05, **p <0.01,***p <0.001), *MFI* mean fluorescence intensity

To unravel the reason for the synergistic effect seen by IFNα-Le and VPA in MOLM-13 cells, we performed phospho-flow exploring the same proteins as described above for IFNα mono-therapy. Altered phosphorylation that could account for the observed synergistic effect was not found for any of the analyzed proteins. Treatment with 1 mM VPA for 15 min resulted in increased acetylation of p53 (acK382), whereas no significant change was induced by IFNα-Le (Fig. 3a, Online Resource Fig. 2C). VPA also induced a slight increase in phospho-ERK1/2 (pT202/pT204) and phospho-p38 (pT180/pY182), similar to the response seen after IFNα-Le treatment, indicating p38 and ERK1/2 as common downstream targets for VPA and IFNα-Le. After 48 h, acetylation of p53 remained to be the main cellular response to VPA treatment, whereas both drugs induced phosphorylation of ERK1/2, p38, p53 (pS15) and Akt (pT308) (Fig. 3b, Online Resource Fig. 2D). The increase in S15 phosphorylation of p53 induced by IFNα-Le indicates that the previously reported induction of p53 by IFNα (Takaoka et al. 2003) may be caused by p53 S15 phosphorylation and not acetylation. A STRING analysis of proteins found to be regulated by IFNα in this study and proteins regulated by VPA in our previous study (Forthun et al. 2012) showed that several proteins were connected, and also showed that proteins found by 2D DIGE interacted with proteins known to be regulated by IFNα (YWHAE and MAPK3(ERK1)/MAPK1(ERK2)/AKT1) (Online Resource Fig. 5).

### Interferon-α gives no survival benefit in MOLM-13^Luc+^ NOD/Scid IL2 γ−/− xenograft mouse

**Fig. 4:**
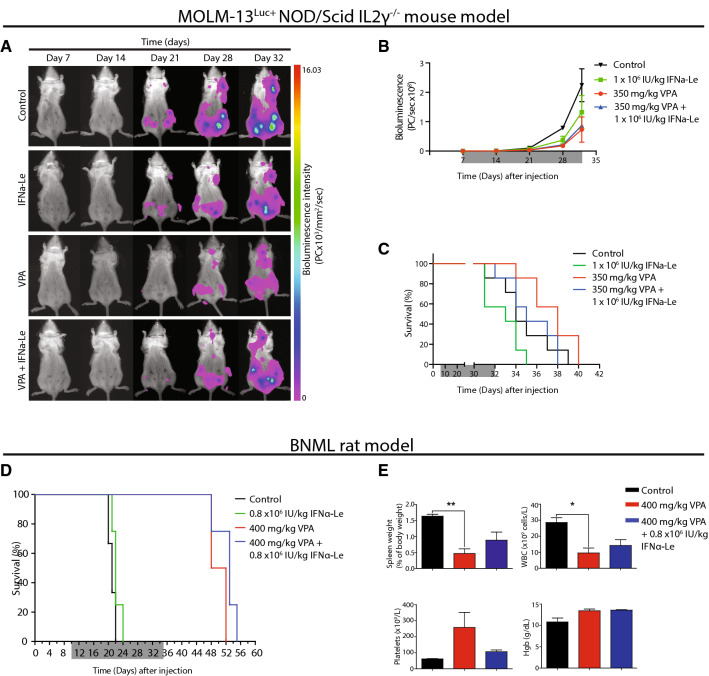
Survival of MOLM-13^Luc+^ NSG mice and BNML rats treated with valproic acid and IFNα-Le. **a** MOLM13^Luc+^ NSG mice (*n* = 2) were imaged weekly after inoculation with 5 million MOLM13^Luc+^ cells. Representative images shows leukemic cell infiltrates day 21 for control and IFNα-Le-treated mice. **b** Total photon counts (ventral) shows lower tumor burden in VPA-treated mice (*n* = 2) compared to other treatments. **c** MOLM-13^Luc+^ NSG mice show significantly increased survival by VPA mono- and combination treatment compared to IFNα-Le-treated mice (*n* = 7). **d** BNML rats showed significantly increased survival by VPA treatment (*n* = 4). Treatment period is indicated in grey. **e** Blood samples and spleens were harvested at humane endpoint (paired *t* test *p = 0.04, **p = 0.01). Samples from IFNα-Le mono-therapy could not be obtained

To further explore the observed in vitro synergistic effects of VPA and IFNα, we used the MOLM-13^Luc+^ NOD/Scid IL2 γ−/− xenograft mouse model. Tumor load evaluation by bioluminescent imaging showed that control mice and mice treated with IFNα-Le (1 × 10^6^ IU/kg) developed tumors in femurs and lymph nodes after 21 days, whilst animals treated with VPA showed detectable tumors 7 days later (Fig. 4a). At day 32, mice treated with VPA (350 mg/kg) showed the lowest tumor burden. Control mice had higher tumor burden compared to IFNα-Le-treated mice (Fig. 4b), but IFNα-Le-treated mice developed hind limb paralysis earlier than other treatment groups. They did, however, not have significantly reduced survival compared to control mice (*p *= 0.118). VPA-treated and VPA/IFNα-Le combination treated mice had significantly longer survival compared to mice treated with IFNα-Le as monotherapy (*p *= 0.0008 and 0.0294, respectively) (Fig. 4c). Necropsy revealed tumor infiltration in lymph nodes and ovaries but no signs of splenomegaly.

### VPA treatment significantly increases survival in immune-competent BN myeloid leukemia rats

The anti-leukemic effects of IFN-α are attributed both to a direct action on AML cells and an indirect effect through immune activation (Anguille et al. 2011). As the MOLM-13^Luc+^ NOD/Scid IL2 γ−/− model is lacking a functional immune system, we further investigated whether the synergistic apoptotic effect observed in MOLM-13 cells could be reproduced in vivo using the immune-competent BNML rat model. Control rats and rats treated with IFNα-Le (0.8 × 10^6^ IU/kg) mono-therapy rapidly presented with hunched posture and paralysis of hind limbs due to the accumulation of leukemic blasts in the bone marrow, and showed median survival of 21 and 22 days, respectively (Fig. 4d). Animals treated with VPA (400 mg/kg), both as mono-therapy and in combination with IFNα-Le, showed no signs of disease during the treatment period and had consistently lower spleen size, white blood cell counts and higher number of platelets (Fig. 4e) compared to control animals. Rats treated with VPA alone and in combination with IFNα-Le also showed significantly longer survival compared to control rats (*p *= 0.01) and compared to IFNα-Le monotherapy (*p *= 0.007). Combining IFNα-Le and VPA (median survival 53 days) gave a slight but non-significant prolonged survival (*p *= 0.07) compared to VPA alone (median survival 50 days), whereas no survival benefit was seen for IFNα-Le mono-therapy. The dose of IFNα-Le used in both the rat and mouse model was slightly higher than the dose chosen for a cutaneous melanoma study (Stadler et al. 2006), but is in line with the current practice for IFNα-2b treatment of chronic myeloid leukemia and chronic hepatitis B (Online Resources).

### Single cell mass cytometry analysis of PBMCs from AML patients and healthy donors

**Fig. 5:**
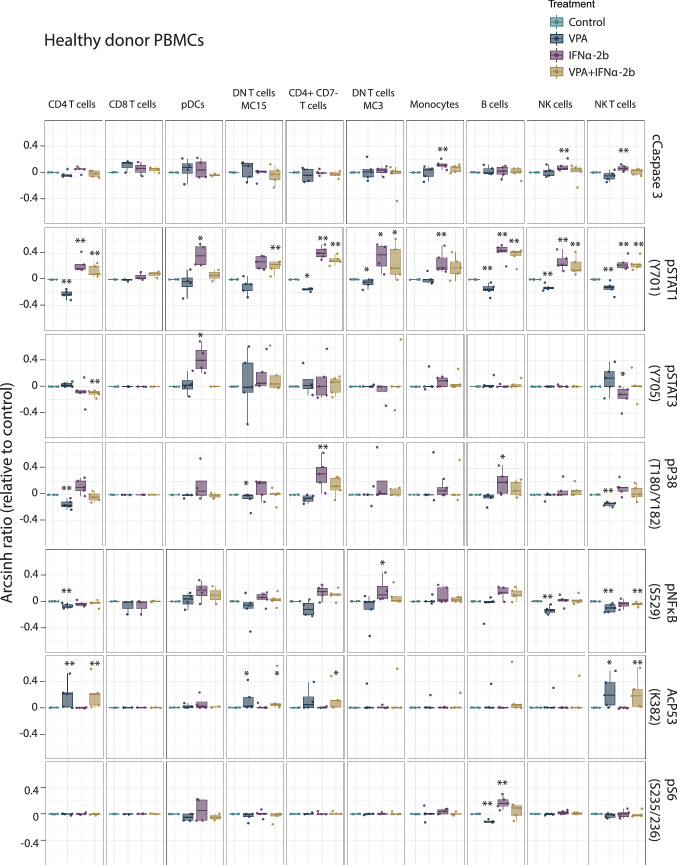
Signaling pathways altered by IFNα-2b and VPA in healthy PBMCs. PBMCs from healthy donors treated with IFNα-2b and VPA and combination IFNα-2b/VPA for 48 h ex vivo were evaluated by CyTOF to investigate alterations in intracellular signaling pathways in defined cell subsets. Data are presented as arcsinh ratio relative to control. Statistics are based on treated cells compared to control. Kruskal–Wallis *H* test *p ≤ 0.05, **p ≤ 0.01

**Fig. 6:**
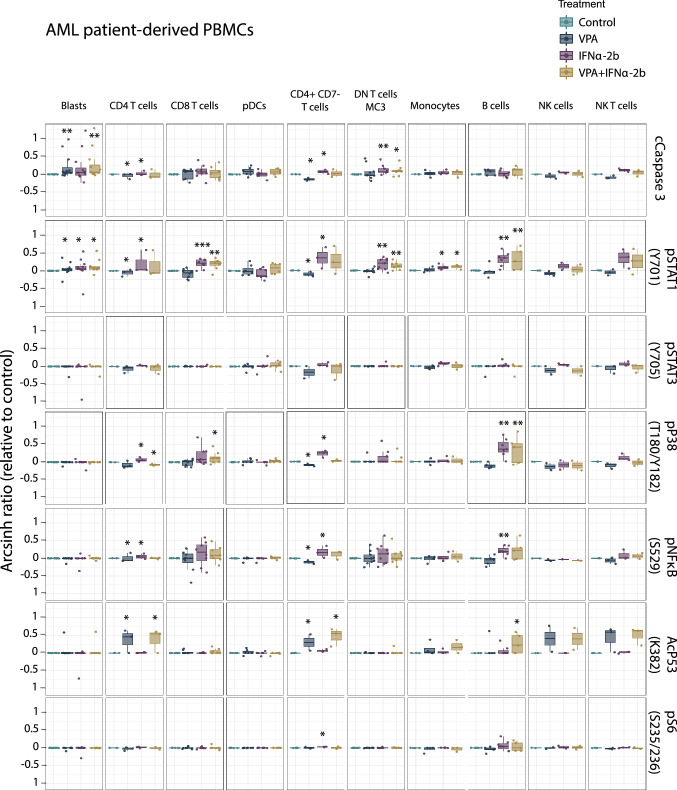
Signaling pathways altered by IFNα-2b and VPA in AML patient-derived PBMCs. PBMCs from AML patients treated with IFNα-2b and VPA and combination IFNα-2b/VPA for 48 h ex vivo were evaluated by CyTOF to investigate alterations in intracellular signaling pathways in defined cell subsets. Data are presented as arcsinh ratio relative to control. Statistics is based on treated cells compared to control. Kruskal–Wallis *H* test *p ≤ 0.05, **p ≤ 0.01

To assess whether the signaling and anti-apoptotic effects observed by IFNα treatment in MOLM-13 was a cell line-specific effect, we treated 12 AML patient and five healthy donor PBMC samples using the same therapy combination (48 h). Using cleaved caspase-3 (cCaspase 3) as a surrogate for apoptosis detection, we found that the two drugs affected healthy donor (Fig. 5) and AML patient PBMCs (Fig. 6) differently. IFNα-2b was the only drug to significantly increase cleaved caspase-3 in healthy donor cells (monocytes; *p* = 0.007, natural killer (NK) cells; *p* = 0.007, NK T-cells; *p* = 0.007). For AML patients, the blast population had significantly increased cleaved caspase-3 by VPA (VPA; *p* = 0.003, VPA/IFNα-2b; *p* = 0.0002). In CD4^+^CD7^−^ T cells from AML patients VPA decreased (*p* = 0.030) and IFNα-2b increased cleaved caspase-3 levels (*p* = 0.017), whereas IFNα-2b gave increased caspase-3 levels in double-negative (DN) T cells (IFNα-2b; *p* = 0.003, VPA/IFNα-2b; *p* = 0.038). No synergistic effects of apoptosis induction were observed by combination therapy in healthy or AML samples.

VPA was found to significantly induce acetylation of p53 (K382) and IFNα-2b to significantly induce phosphorylation of STAT1 (Y701) and STAT3 (Y705) both in healthy donor and AML PBMCs (Figs. 5 and 6), validating the finding made by flow cytometry in the MOLM-13 cell line (Fig. 3). NPM1 wild type patients had higher levels of pSTAT1 after IFNα-2b treatment compared to mutated patients (DN T cells; *p *= 0.049) (Online Resource Fig. 6).

**Fig. 7:**
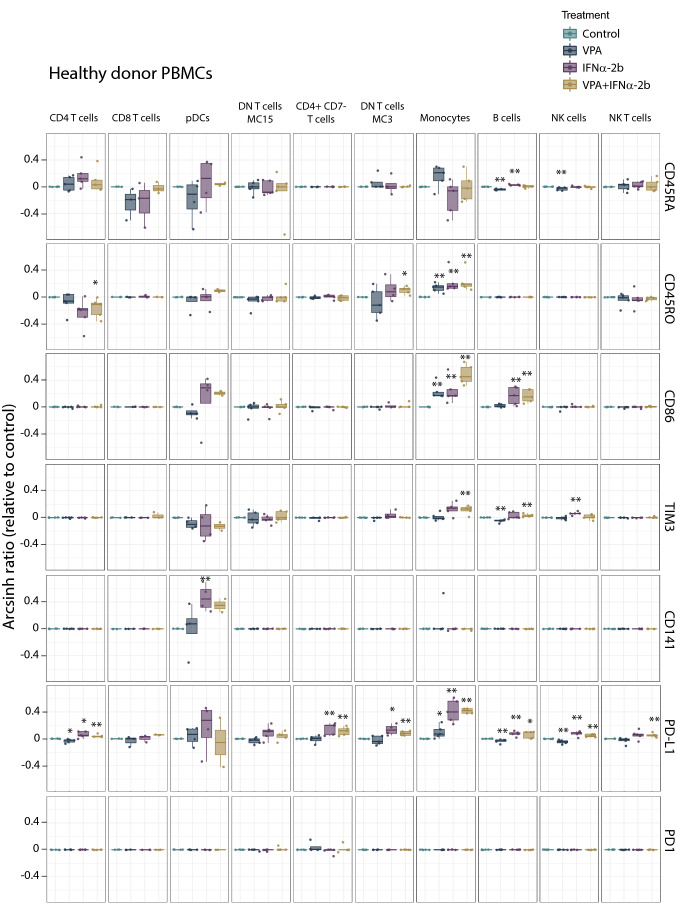
Immune activation markers altered by IFNα-2b and VPA in healthy PBMCs. PBMCs from healthy donors treated with IFNα-2b and VPA and combination IFNα-2b/VPA for 48 h ex vivo were evaluated by CyTOF to investigate alterations in immune activation markers in defined cell subsets. Data are presented as arcsinh ratio relative to control. Statistics are based on treated cells compared to control. Kruskal–Wallis *H* test *p ≤ 0.05, **p ≤ 0.01

**Fig. 8:**
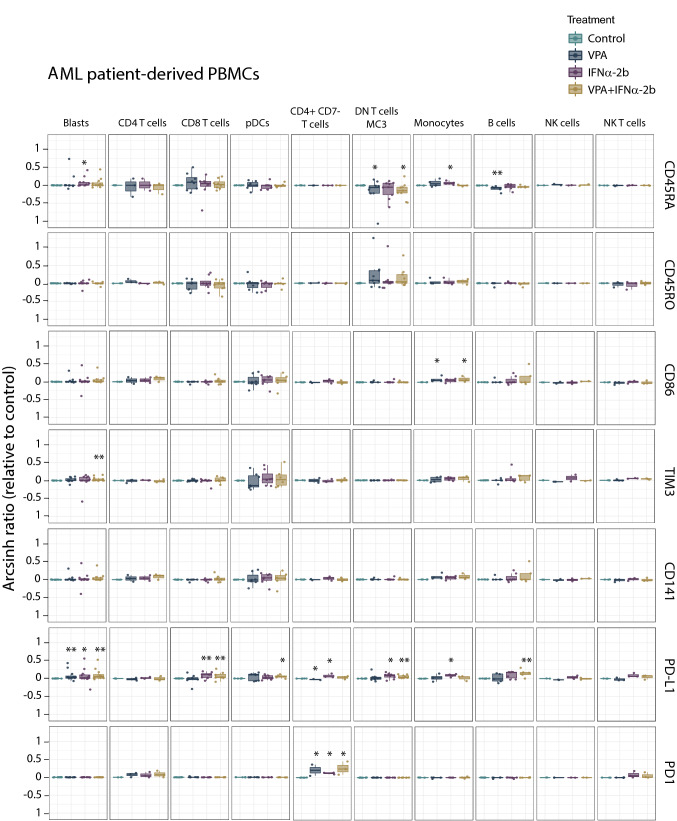
Immune activation markers altered by IFNα-2b and VPA in AML patient-derived PBMCs. PBMCs from AML patients treated with IFNα-2b and VPA and combination IFNα-2b/VPA for 48 h ex vivo were evaluated by CyTOF to investigate alterations in immune activation markers in defined cell subsets. Data are presented as arcsinh ratio relative to control. Statistics are based on treated cells compared to control. Kruskal–Wallis *H* test *p ≤ 0.05, **p ≤ 0.01

Investigating the immune-modulating effects of both VPA and IFNα-2b (see Online Resource Table 2 for antibody panel), we observed that IFNα-2b treatment of healthy PBMCs (Fig. 7) up-regulated CD141 on plasmacytoid dendritic cells (pDCs; *p* = 0.021), and up-regulated PD-L1 (CD4^+^ T cells; *p* = 0.025, CD4^+^CD7^−^ T cells; *p* = 0.007, DN T cells; *p* = 0.011, monocytes; *p* = 0.007, B cells; *p* = 0.007 and NK cells; *p* = 0.007), CD45RO (monocytes; *p* = 0.007), CD86 (monocytes; *p* = 0.007, B cells; *p* = 0.007) and TIM3 (NK cells; *p* = 0.007). For AML patient PBMCs (Fig. 8), no significant change in levels of CD141, CD45RO, CD86 or TIM3 was found by IFNα-2b treatment. CD45RA was, however, slightly increased in blasts (*p* = 0.034) and monocytes (*p* = 0.021). PD1 (CD4^+^CD7^−^ T cells; *p* = 0.017) and PD-L1 (blasts; *p* = 0.05, CD8^+^ T cells; *p* = 0.006, CD4^+^CD7^−^ T cells; *p* = 0.017, DN T cells; *p* = 0.038, monocytes; *p* = 0.021) were also increased by IFNα-2b treatment. Comparing healthy donor and AML PBMCs after IFNα-2b treatment showed that healthy donors had monocytes with stronger induction of PD-L1 (*p* = 0.014), CD86 (*p* = 0.05) and CD45RO (*p* = 0.049) in addition to pDCs with higher levels of CD141 (*p* = 0.023), whereas AML patients had CD4^+^CD7^−^ T cells with increased levels of PD1 (*p* = 0.022). Subdividing patients according to karyotype (Online Resource Fig. 7) showed that patients with normal karyotype had higher levels of PD-L1 compared to patients with complex karyotype (pDCs); *p* = 0.032) after IFNα-2b treatment. B cells in NPM1 mutated patients (Online Resource Fig. 6) had higher levels of CD45RA compared to wild type patients (*p* = 0.032). No significant changes were found by IFNα-2b monotherapy between FLT3 internal tandem duplication (ITD) mutated and wild type patients (Online Resource Fig. 9).

## Discussion

The phosphoproteome analysis identified the acetyl transferase protein NAA10 as selectively down regulated by IFNα-Le and a potential overlapping signal pathway with the histone deacetylase inhibitor VPA. Knock-down of NAA10 has been found to increase apoptosis and increase the sensitivity to daunorubicin in vitro (Arnesen et al. 2006). Furthermore, mutations in the auto-acetylation site of NAA10 inhibit lung tumor xenograft growth in vivo (Seo et al. 2010). YWHAE and PKM2 expression were also induced by IFNα-Le, and knockdown of these proteins has been shown to result in increased invasion, migration and proliferation in gastric cancer cell lines (Leal et al. 2016), and inhibition of drug-induced differentiation in leukemic K562 cells (Chaman et al. 2015). Additionally, mRNA expression of YWHAE, ARMC6, RAB11B, P4HB, SNRPA and ANP32A is down-regulated and CAPZB up-regulated by VPA in AML cell lines (Rucker et al. 2016), further supporting the existence of overlapping signaling pathways for IFNα and VPA. STRING pathway analysis of proteins regulated by IFNα and VPA (Forthun et al. 2012) also showed several interactions (Online Resource Fig. 5) determined experimentally.

Previous case reports indicate that secondary AML transformed from essential thrombocytosis or myelofibrosis particularly benefit from IFNα therapy (Berneman et al. 2010; Dagorne et al. 2013). In the light of the biological and molecular heterogeneity in AML (Dohner et al. 2017), this may suggest that a particular AML subset is sensitive for IFNα. This was, however, not the case for our patient cohort, although the number of individuals was limited. Importantly, immediate and rapid progressing disease has been described in acute lymphoblastic leukemia patients treated with lower doses of IFNα (Ochs et al. 1986) and in vitro testing of AML cells has indicated that approximately a third of the patient samples responded with increased clonogenicity when treated with lower doses IFNα (Ludwig et al. 1983). Our study showed increased in vitro activation of UPR by IFNα-induced phosphorylation of AK2 and HSPH1 in MOLM-13 cells. Increased UPR has been shown to promote faster tumor growth and resistance to common anticancer drugs in xenograft mouse models (Bi et al. 2005; Spiotto et al. 2010). Therefore, moderate or low doses IFNα should be used with care in AML until we know predictive markers for therapy response, like tumor burden and molecularly defined IFNα-sensitive subtypes of AML.

The anti-tumor effect of IFNα in combination with VPA has been suggested experimentally in other cancers (Stadler et al. 2006; Iwahashi et al. 2011; Hudak et al. 2012). We observed that IFNα was synergistic in combination with VPA in MOLM-13 cells in vitro. A similar in vitro and in vivo synergism has been demonstrated combining VPA with the small molecule MDM2 inhibitor nutlin-3 in the MOLM-13^Luc+^ xenograft model (McCormack et al. 2012). However, IFNα-Le treatment of the MOLM-13^Luc+^ xenograft model indicated no survival benefit, and the same was found in the immune competent BNML model. Previous studies treating the aggressive BNML model with interferon-inducing BCG demonstrated a similar lack of survival benefit in the monotherapy arm (Hagenbeek and Martens 1983). Furthermore, low tumor burden is a prerequisite for IFNα anti-tumor response (Eggermont et al. 2012), and we cannot exclude that the mouse and rat models used in this study may have exceeded the tumor burden accessible for beneficial IFNα therapy.

Whereas the lack of anti-leukemic effect of IFNα in the mouse model used in our study could also be due to the absence of important immune cells needed for an effective DC effect against AML cells (Ito et al. 2002), the BNML rat model has an intact immune system. The lack of in vivo potency of the VPA and IFNα-Le combination in this model could be a result of reduced activity of human IFNα-Le in rats. However, we did find 2000 IU/mL IFNα-Le to increase pSTAT1 (Y701) in BNML derived IPC-81 cells (Online Resource Fig. 4), suggesting that human IFNα could be reactive also in BNML rats. Furthermore, activation of rat IFNα receptors by human IFNα is supported by reports of reduced rat endometriosis by human IFNα (Altintas et al. 2008) and in vivo interferon-induced metallothionein (Guevara-Ortiz et al. 2005).

It is well established that IFNα activates DCs, T cells and NK cells, and thus contributes to the generation of a potent anti-leukemic immune response (Zhang et al. 2005; Watanabe et al. 2006; Korthals et al. 2007; Willemen et al. 2015). Investigating immune regulators in healthy and AML patient-derived PBMCs revealed that all cellular subsets apart from CD8^+^ and DN T cells responded by one or more markers after IFNα-2b treatment in healthy donor PBMCs. For AML-derived PBMCs, however, no response was seen in pDCs, NK or NK T cells. We also observed that PBMCs from AML patients had different immune-associated responses to IFNα-2b compared to healthy donor PBMCs (Online Resource Fig. 8). Particularly, lack of activation of the differentiation markers CD141, CD45RO and CD86 in AML patient monocytes and pDCs could indicate that patients with AML have an inaccessible immune system where cell subsets stay unresponsive to activating stimuli. The absence of pSTAT1 (Y701) induction in AML-derived pDCs, and not healthy pDCs, further supports the inability of these cells to respond to IFNα. The up-regulation of CD141 in response to IFNα treatment in healthy pDCs was importantly not observed in AML-derived DCs. CD141^+^ DCs are known to induce differentiation of IL-4- and IL-13-producing CD4^+^ T cells, thereby guiding the adaptive immune response (Yu et al. 2014). Thus, the lack of DC activation in AML samples could explain the lack of response to IFNα monotherapy in AML patients. Increased CD86 promotes myeloid differentiation and suppresses cell proliferation (Fang et al. 2017). AML patients positive for CD86 have been suggested to be candidates for immunotherapy (Re et al. 2002), however, this marker was not activated in AML patients. Neither was CD45RO, whose presentation on lymphocytes in adult T cell leukemia patients is correlated with improved prognosis (Suzuki et al. 1998). Thus, our overall results could indicate that there is a combined loss of the differentiation potential and lack of immune activation in the investigated AML patients, suggesting that these patients would not benefit from IFNα-monotherapy.

## Conclusion

IFNα-2b and IFNα-Le have different effects on the regulation of phospho-protein expression as discovered by 2D DIGE proteomic analysis and phospho-flow, and IFNα combined with VPA induced cell death synergism in vitro. The absence of monocyte and pDC activation by IFNα ex vivo could explain the lack of an in vivo anti-leukemic effect, and the therapeutic effect of IFNα may potentially be enhanced by removing this inherent block of activation in healthy immune subsets in AML patients. This needs to be addressed in future studies that take into consideration the complex tumor-host interactions in AML.

## Electronic supplementary material

Below is the link to the electronic supplementary material.

Supplementary material 1 (PDF 1191 kb)

Supplementary material 2 (PDF 676 kb)

Supplementary material 3 (PDF 257 kb)

Supplementary material 4 (PDF 428 kb)

Supplementary material 5 (PDF 8535 kb)

Supplementary material 6 (PDF 2143 kb)

Supplementary material 7 (PDF 1588 kb)

Supplementary material 8 (PDF 2171 kb)

Supplementary material 9 (PDF 1955 kb)

Supplementary material 10 (DOCX 73 kb)

## Abbreviations

IFNα: Interferon alpha
VPA: Valproic acid
AML: Acute myeloid leukemia
HSCT: Hematopoietic stem cell transplantation
MRD: Minimal residual disease
ATRA: All-*trans* retinoic acid
BNML: Brown Norwegian myeloid leukemia
NSG: NOD/Scid IL2 γ−/−
RT: Room temperature
PI: Propidium Iodide
IMAC: Immobilized affinity chromatography
2D DIGE: Two dimensional difference gel electrophoresis
PFA: Paraformaldehyde
PBMC: Peripheral blood mononuclear cell
DC: Dendritic cell
pDC: Plasmacytoid dendritic cell
DN T cell: Double negative T cell
NK: Natural killer
MFI: Mean fluorescence intensity
UPR: Unfolded protein response
ITD: Internal tandem duplication
