# Supplementary material for: Modulation of phospho-proteins by interferon-alpha and valproic acid in acute myeloid leukemia
Source: J Cancer Res Clin Oncol. 2019 May 20;145(7):1729–49. doi: 10.1007/s00432-019-02931-1 (PMC6571093; doi:10.1007/s00432-019-02931-1)

Metaclusters defined by Phenograph:

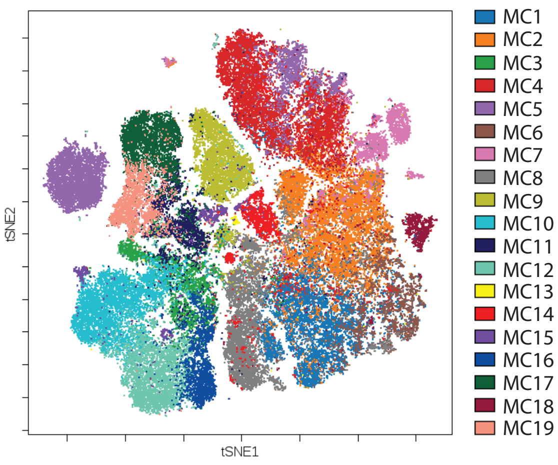

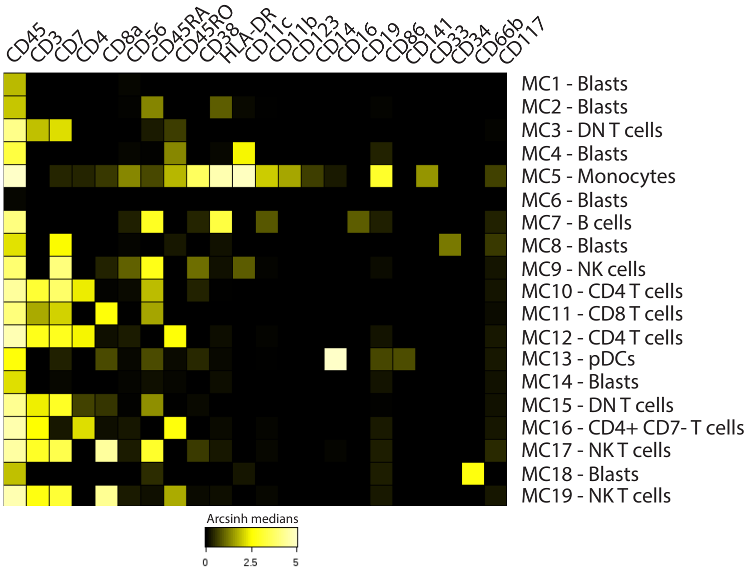

Metaclusters manually concatenated:

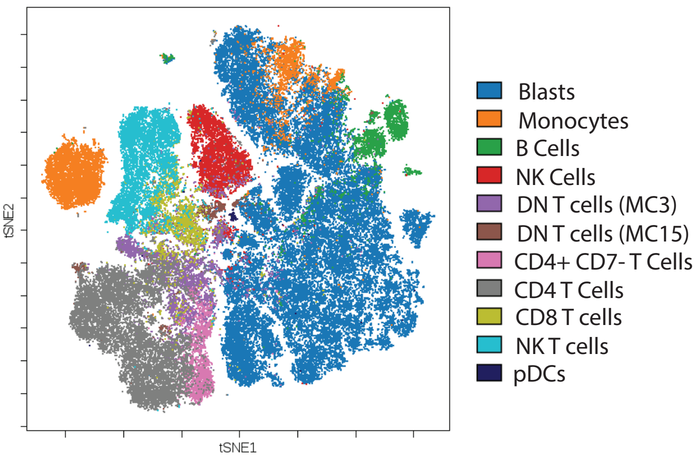

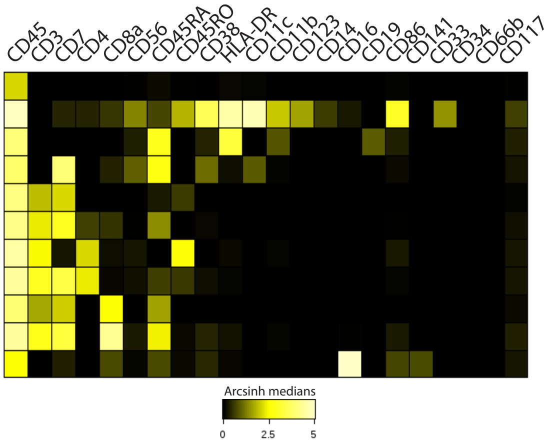

Immunophenotype blasts in individual patients:

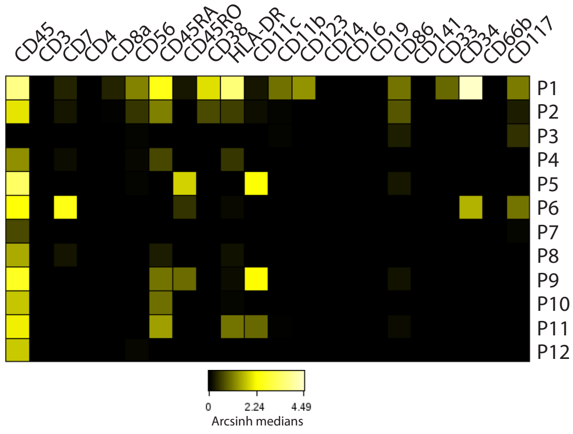

Supplement: Supplementary file 1 — Supplementary material 1 (PDF 1191 kb) [file 432_2019_2931_MOESM1_ESM.pdf]
