# Supplementary figures and images for: Modulation of phospho-proteins by interferon-alpha and valproic acid in acute myeloid leukemia
Source: J Cancer Res Clin Oncol. 2019 May 20;145(7):1729–49. doi: 10.1007/s00432-019-02931-1 (PMC6571093; doi:10.1007/s00432-019-02931-1)

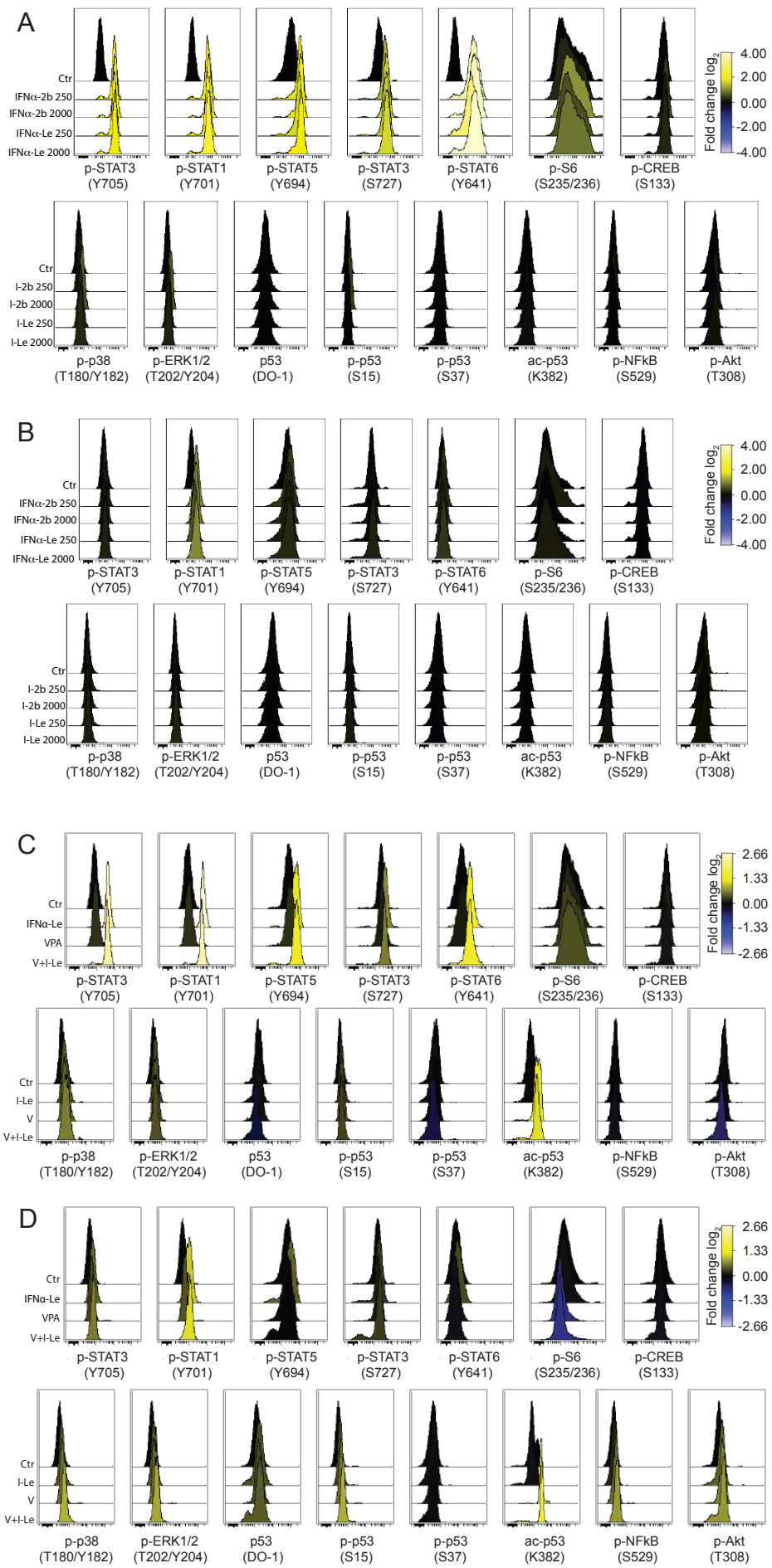

Supplement: Supplementary file 2 — Supplementary material 2 (PDF 676 kb) [file 432_2019_2931_MOESM2_ESM.pdf]

**A**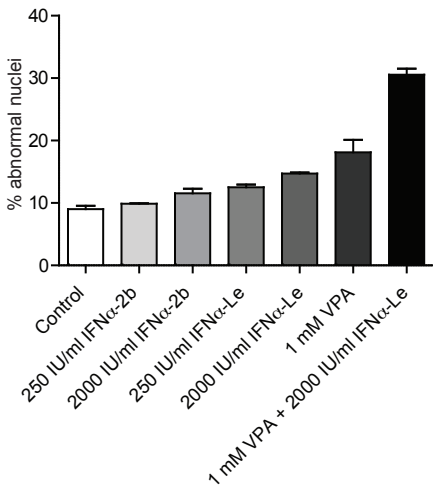**B**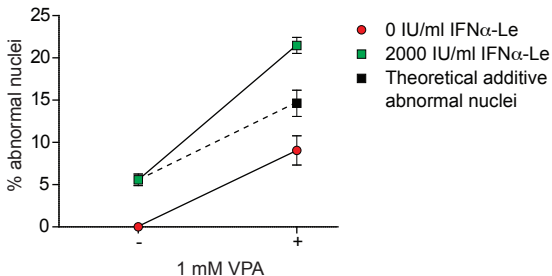

Supplement: Supplementary file 3 — Supplementary material 3 (PDF 257 kb) [file 432_2019_2931_MOESM3_ESM.pdf]

**A**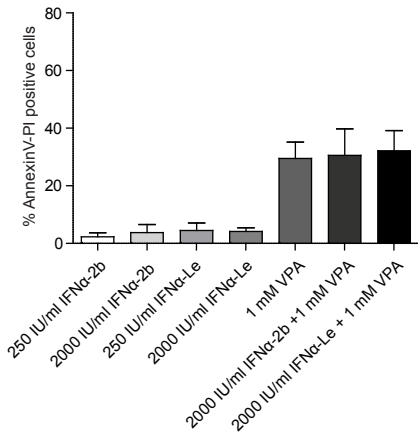**B**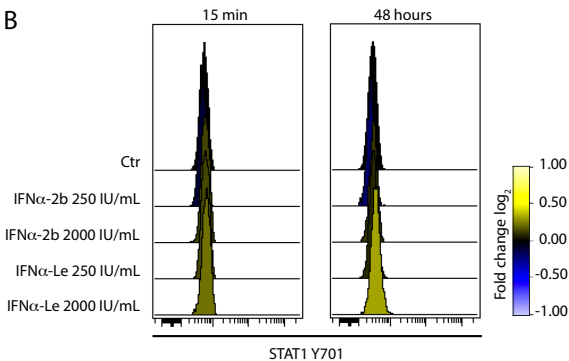

Supplement: Supplementary file 4 — Supplementary material 4 (PDF 428 kb) [file 432_2019_2931_MOESM4_ESM.pdf]

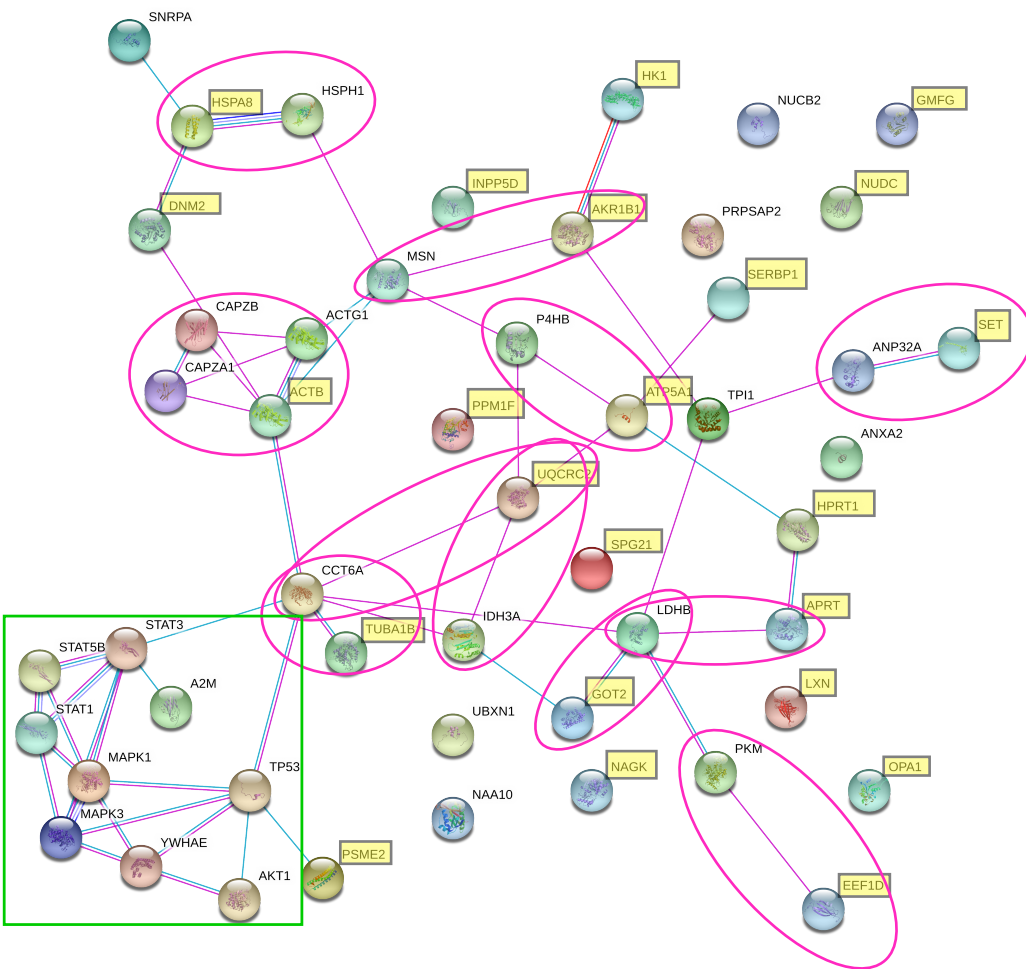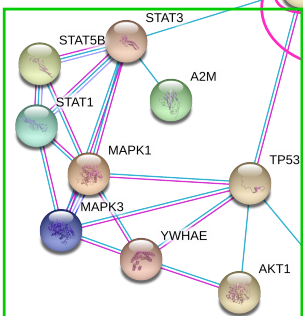

Supplement: Supplementary file 5 — Supplementary material 5 (PDF 8535 kb) [file 432_2019_2931_MOESM5_ESM.pdf]

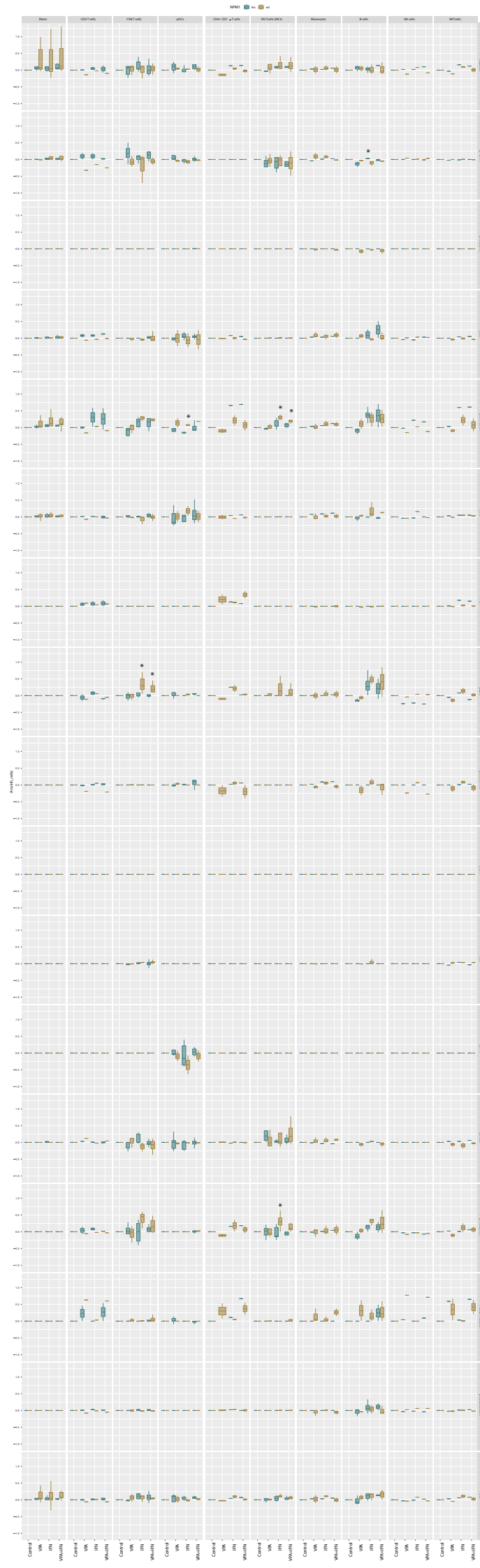

Supplement: Supplementary file 6 — Supplementary material 6 (PDF 2143 kb) [file 432_2019_2931_MOESM6_ESM.pdf]

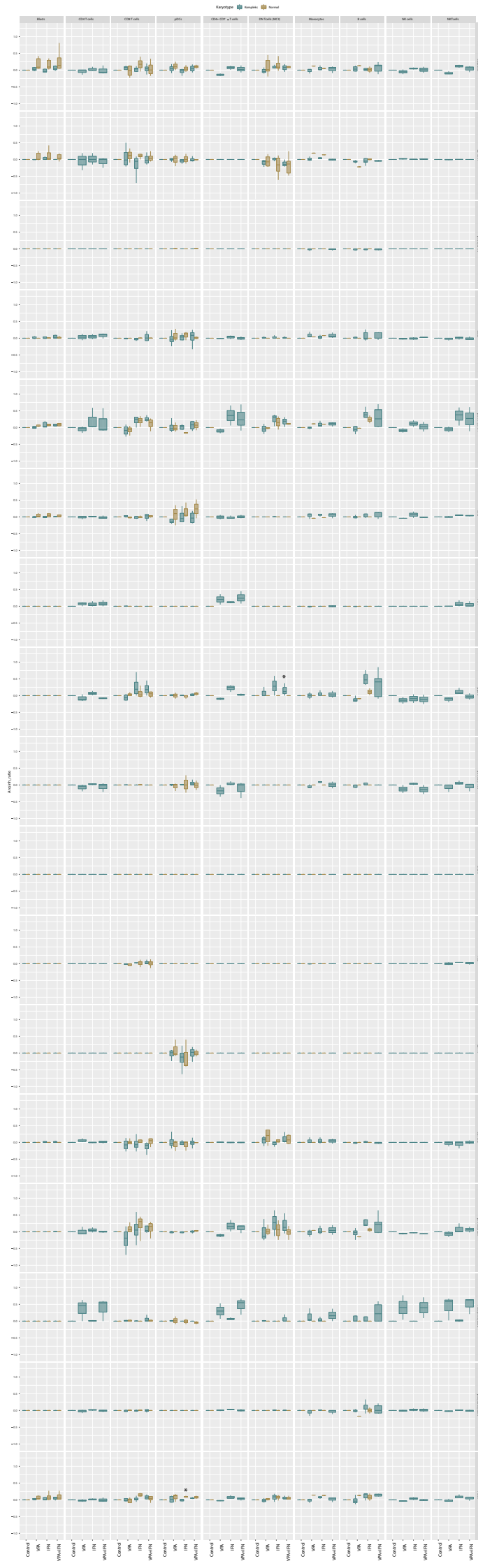

Supplement: Supplementary file 7 — Supplementary material 7 (PDF 1588 kb) [file 432_2019_2931_MOESM7_ESM.pdf]

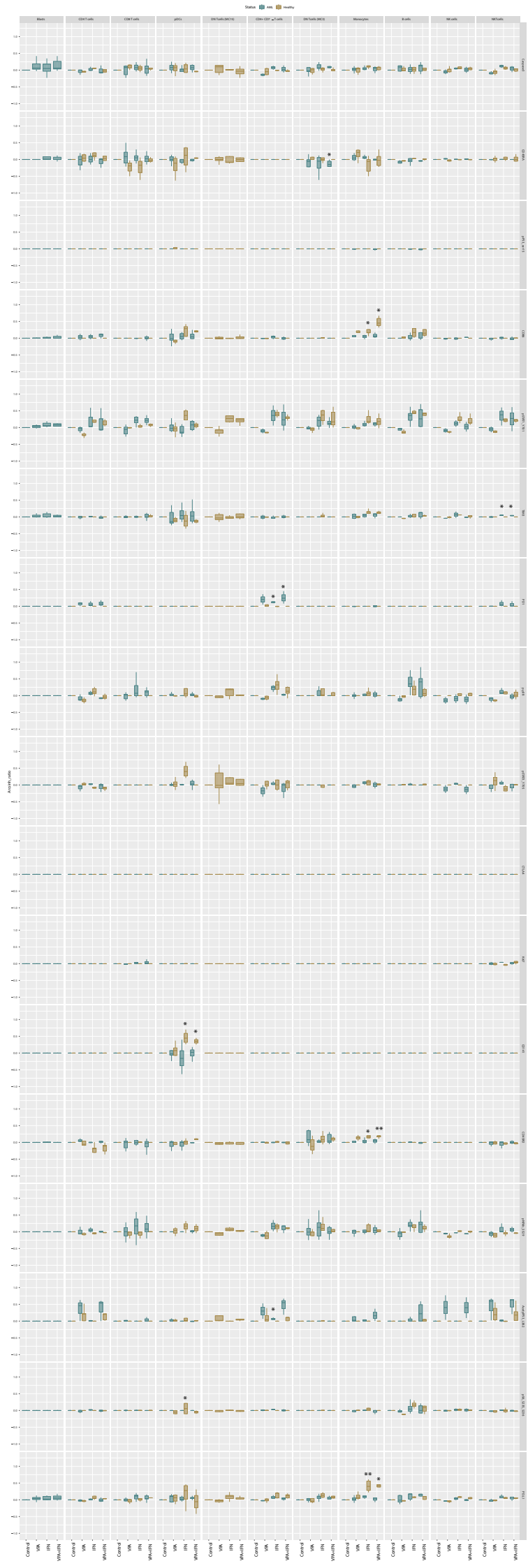

Supplement: Supplementary file 8 — Supplementary material 8 (PDF 2171 kb) [file 432_2019_2931_MOESM8_ESM.pdf]

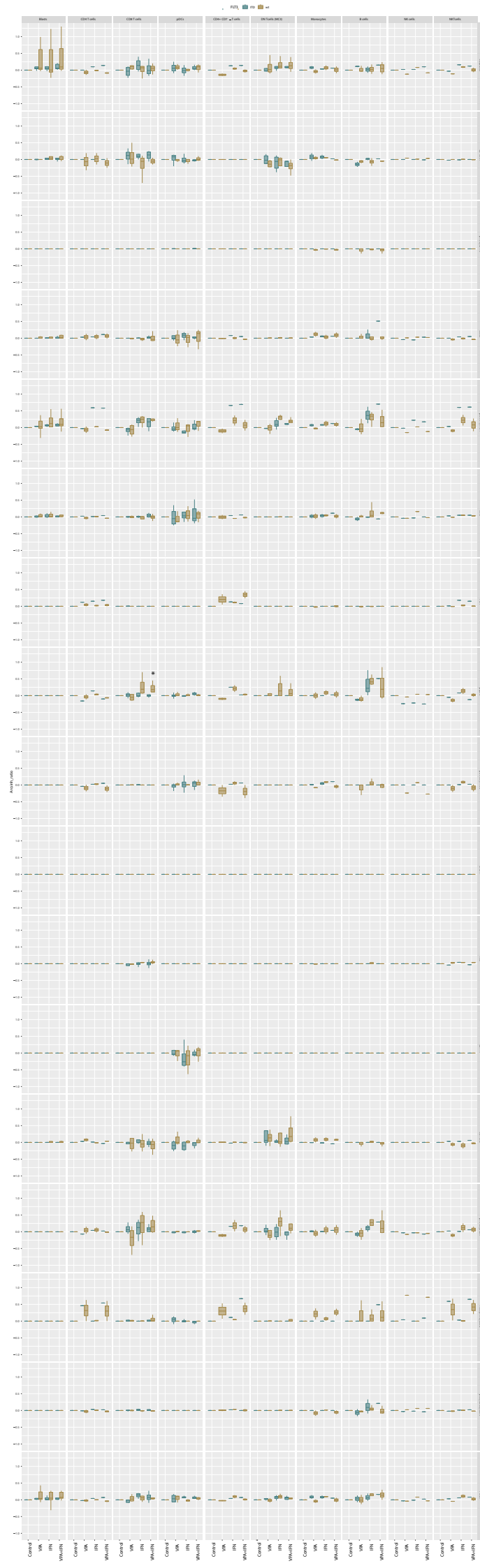

Supplement: Supplementary file 9 — Supplementary material 9 (PDF 1955 kb) [file 432_2019_2931_MOESM9_ESM.pdf]
