# Supplementary material for: Modulation of phospho-proteins by interferon-alpha and valproic acid in acute myeloid leukemia
Source: J Cancer Res Clin Oncol. 2019 May 20;145(7):1729–49. doi: 10.1007/s00432-019-02931-1 (PMC6571093; doi:10.1007/s00432-019-02931-1)
Supplement: Supplementary file 10 — Supplementary material 10 (DOCX 73 kb) [file 432_2019_2931_MOESM10_ESM.docx]

**Modulation of phospho-proteins by interferon-alpha and valproic acid in acute myeloid leukemia**

**Rakel Brendsdal Forthun^1^, Monica Hellesøy^2^, André Sulen^1^, Reidun Kristin Kopperud^1^, Gry Sjøholt^3,^ Øystein Bruserud^2, 4^, Emmet McCormack^1, 2^ and Bjørn Tore Gjertsen^1, 2^**

^1^ Centre for Cancer Biomarkers (CCBIO), Department of Clinical Science, Precision Oncology Research Group, University of Bergen, Bergen, Norway; ^2^ Department of Internal Medicine, Hematology Section, Haukeland University Hospital, Bergen, Norway; ^3^ Department of Biomedical Laboratory Sciences and Chemical Engineering, Bergen University College, Bergen, Norway; ^4^ Department of Clinical Science, Faculty of Medicine and Dentistry, University of Bergen, Bergen, Norway

**Material and methods**

# *Cell culture*

# MOLM-13 (DSMZ, Braunschweig, Germany) was grown in RPMI 1640 (Gibco, Invitrogen, Paisley, UK) supplemented with 10% fetal bovine serum gold (PAA Laboratories GmbH, Pasching, Austria), streptomycin (5mg/mL), penicillin (5U/mL) and L-glutamine (2mM) (all from Sigma Aldrich, Oslo, Norway) at 37ºC with 5% CO_2_ in a humidified incubator. The rat BNML cell line IPC-81 (obtained from Dr. Michel Lanotte ([Lacaze, Gombaud-Saintonge et al. 1983](#_ENREF_4))) was grown in DMEM (Gibco, Invitrogen) supplemented with 10% horse serum (PAA Laboratories GmbH), streptomycin, penicillin and L-glutamine as described above. Cells were harvested for phospho-flow, Hoechst staining and Annexin-V/propidium iodide analysis as described below, as well as cell lysis and phosphoprotein purification by immobilized metal affinity chromatography as previously described ([Forthun, Sengupta et al. 2012](#_ENREF_3)).

# *Phospho-flow cytometry*

# Fixed, permeabilized and barcoded samples were washed with 0.5% BSA in PBS and re-suspended in 0.5% BSA in PBS with 200 µg/mL human IgG (Octagam, Octapharma AG, Lachen, Switzerland) for blocking. Samples were split in equal parts for staining using six different panels of intracellular antibodies (Supplementary Table 1). Following washing with PBS, the samples were re-suspended in PCS and analyzed by flow cytometry. The data were collected on a FACS Fortessa flow cytometer (BD Biosciences, San Diego, CA, USA), analysis was carried out in CytoBank ([www.cytobank.org](http://www.cytobank.org)). To analyze only live cells, a strict gate was set in forward- and side-scatter dot plot, including the live population only.

# *Cell viability and cell death assays*

For cell viability investigation, treated cells were washed in 2 ml PBS and centrifuged at 400 g, followed by incubation for 15 minutes at room temperature in 50 μl binding buffer containing 2.5 % Annexin-V Alexa Fluor 488. Prior to analysis the samples were added 200 μl binding buffer with propidium iodide (final concentration 0.2 μg/mL). Data were acquired on a BD Accuri C6 flow cytometer (BD Bioscience, San Jose, CA, USA) and analyzed using Flow Jo (Tree Star, Inc., Ashland, OR, USA). To investigate cell death, cells were stained with 10 µg/ml Hoechst 33342 DNA stain (Calbiochem, Merck KGaA, Darmstadt, Germany) for one hour at RT prior to scoring abnormal nuclei by examining nuclear morphology of at least 300 cells in triplicate by epifluorescence microscopy (Leica DM IRB, Leica Microsystems, Mannheim, Germany). The numbers of dead cells are displayed relative to the control. Statistical significance was determined using the Student’s unpaired, two tailed T-test for individual treatments, or two-way ANOVA (GraphPad) for synergism testing (*p* < 0.05).

*Two-dimensional differential gel electrophoresis and mass spectrometry*

Phosphoprotein samples were covalently labeled with fluorescent CyDyes (GE Healthcare) in a minimal labeling reaction (400 pmol dye:50 µg protein) as described previously ([Alban, David et al. 2003](#_ENREF_1)). Pooled, labeled phosphoprotein samples were cup-loaded on pH 3-11 DryStrip Immobiline gel strips (GE Healthcare) and isoelectric focusing was performed by the specifications; 150 V Step 3 hours, 300 V Step 3 hours, 1000 V Gradient 6 hours, 8000 V Gradient 2 hours, 8000 V Step 3 hours. Focused strips were equilibrated (6 M urea, 2% SDS, 75 mM Tris-HCl, pH 6.8, 30% glycerol) supplemented with 15 mg/ml dithiothreitol for 15 min at room temperature, followed by 45 mg/ml iodoacetamide for 10 min. Second dimension gel electrophoresis was performed on 26 x 20 cm 10% Ettan DALTsix gels casted in the lab and run as described by manufacturers. Gels were run at 20°C, 10 mA/gel, 1 W/gel, 150 V for 1 hour, 12 mA/gel, 2 W/gel, 150 V for 17 hours, and finally 400 mA, 72 W, 500 V until the bromophenol blue front was at the border of the gel plates. The gels were scanned using a Typhoon TRIO Variable Mode Imager (GE Healthcare) and spots representing phosphoproteins were co-detected and quantified using the Differential In-gel Analysis module of the DeCyder 7.0 software (GE Healthcare). Protein statistics (Students unpaired t-test, p < 0.05) was performed in the Biological Variation Analysis module, excluding proteins present in less than 75% of the spotmaps, and proteins with lower fold change than 1.3. Preparative gels were run using 500 µg MOLM-13 lysate and labeled with SYPRO Ruby gel staining (Bio-Rad, [Hercules, California](http://en.wikipedia.org/wiki/Hercules,_California), [US](http://en.wikipedia.org/wiki/United_States)A) overnight according to the producer’s recommendations. Gels were stored in 10% ethanol at 4ºC until automatic spot picking by Ettan Spot Picker as described by the producers (GE Healthcare). Protein identification was performed using the LTQ-Orbitrap XL (Thermo Scientific, Waltham, MA, USA) as described ([Bull, Fargestad et al. 2010](#_ENREF_2)). A minor adjustment was made increasing the starting solvent from 0 to 7% of solvent B (90% acetonitrile with 0.1% formic acid).

*In vivo IFNα-Le treatment*

IFNα-Le doses were based on traditional doses available at time of study start for IFNα-2b delivered to patients with chronic hepatitis B (5-10x10^6^ IU, 3 times a week), chronic hepatitis C (3x10^6^ IU, 3 times a week) and chronic myeloid leukemia (4-5x10^6^/m^2^ daily), in addition to a study on cutaneous melanoma using IFNα-Le (3x10^6^ IU, 3 times a week) ([Nair and Jacob 2016](#_ENREF_5)). Using the dose conversion extrapolation by Nair and Jacob ([Nair and Jacob 2016](#_ENREF_5)), the human equivalent dose (HED) of the Brown Norwegian Myeloid Leukemia (BNML) rat model (0.8x10^6^ IU/kg, 3 times a week) was 0.13x10^6^ IU/kg, 3 times a week, whilst the HED of the MOLM-13^Luc+^ mouse model (1x10^6^ IU/kg, 3 times a week) was 0.08x10^6^ IU/kg, 3 times a week.

*BNML rat hematological investigation*

Spleens from the BNML rat model were weighed at humane endpoint, and presented as percentage weight of total body weight. Blood was drawn by terminal heart puncture and hematological parameters were investigated by standard procedures for rat blood by the Laboratory of Clinical Biochemistry, Haukeland University Hospital, Bergen, Norway. Control sample data are from control animals presented in our previous study of BNML ([Forthun, Sengupta et al. 2012](#_ENREF_3)). Rats treated with IFNα-Le monotherapy progressed so rapidly that samples for hematological investigations were not collected. Statistical testing of hematological values and spleen weights were performed by the Student’s two-tailed, unpaired T-test.

*Optical imaging*

Briefly, anesthetized mice were injected intra peritoneally with D-luciferin (150 mg/kg) 10 minutes prior to whole body imaging, performed with the In-Vivo FX Pro molecular imaging system (Carestream Health, Inc., Rochester, NY, USA). The images were acquired over 90 seconds with 2x2 pixels binning. FX Pro images were analyzed using Carestream MI software (Standard Edition, v5.0.6.20, Carestream Health, Inc.)

**Online Resource Table 1. CyTOF antibodies and metals used in panel 1.**

| **Antibody** | **Clone** | **Mass tag** | **Vendor** | **Cat.no** |
| --- | --- | --- | --- | --- |
| ***Barcode:*** |  |  |  |  |
| MBC #1 |  | 102 Pd | Fluidigm | 201060 |
| MBC #2 |  | 104 Pd | Fluidigm |  |
| MBC #3 |  | 105 Pd | Fluidigm |  |
| MBC #4 |  | 106 Pd | Fluidigm |  |
| MBC #5 |  | 108 Pd | Fluidigm |  |
| MBC #6 |  | 110 Pd | Fluidigm |  |
|  |  |  |  |  |
| ***Surface panel 1:*** |  |  |  |  |
| CD45 | Hi30 | 89 Y | Fluidigm | 3089003B |
| CD66b | G10F5 | 141 Pr | BioLegend | 305102 |
| CD117 (cKit) | 104D2 | 143 Nd | Fluidigm | 3143001C |
| CD38 | HIT2 | 144 Nd | Fluidigm | 3144014C |
| CD4 | RPA-T4 | 145 Nd | Fluidigm | 3145001B |
| CD8a | RPA-T8 | 146 Nd | Fluidigm | 3146001B |
| CD20 | 2H7 | 147 Nd | Fluidigm | 3147001B |
| CD16 | 3G8 | 148 Nd | Fluidigm | 3148004B |
| CD123 (IL-3R) | 6H6 | 151 Eu | Fluidigm | 3151001B |
| CD56 (NCAM) | B159 | 155 Gd | Fluidigm | 3155008B |
| CD14 | M5E2 | 160 Gd | Fluidigm | 3160001B |
| CD33 | WM53 | 163 Dy | Fluidigm | 3163023B |
| CD34 | 581 | 168 Er | BioLegend  In-house conjugation | 343531 |
| CD19 | HIB19 | 169 Tm | Fluidigm | 3169011B |
| CD3 | UCHT1 | 170 Er | Fluidigm | 3170001B |
| CD300e/IREM2 | 233810 | 173Yb | R&D Systems  in-house conjugation | MAB2705 |
| HLA-DR | L243 | 174 Yb | Fluidigm | 3174001C |
| CD11b (Mac-1) | Mac-1 | 209 Bi | Fluidigm | 3209003B |
|  |  |  |  |  |
| ***Intracellular panel 1:*** |  |  |  |  |
| Caspase 3 (cleaved) | D3E9 | 142 Nd | Fluidigm | 3142004C |
| pP53 (S15) | 261366 | 149 Sm | R&D Systems  In-house conjugation | MAB18391 |
| pAkt (S473) | D9E | 152 Sm | Fluidigm | 3152005C |
| pSTAT1 (Y701) | 58D6 | 153 Eu | Fluidigm | 3153003C |
| p-p38 (T180/Y182) | D3F9 | 156 Gd | Fluidigm | 3156002C |
| pSTAT3 (Y705) | 4 | 158 Gd | Fluidigm | 3351607 |
| pBAD (S112) | 40A9 | 161 Dy | Fluidigm | 1251506 |
| Ki67 | B56 | 162 Dy | Fluidigm | 3162012C |
| Cyclin B1 | GNS-1 | 164 Dy | Fluidigm | 3153009C |
| pCREB (S133) | 87G3 | 165 Ho | Fluidigm | 3165009A |
| pNFkB (S529) | K10-895.12.50 | 166 Er | Fluidigm | 3166006A |
| Acetyl-P35 (K382) | REA529 | 167 Er | Miltenyi Biotec | 5170614144 |
| pErk1/2 (T202/Y204) | D13.14.4 | 171 Yb | Fluidigm | 3171010C |
| pS6 (S235/236) | N7-548 | 172 Yb | Fluidigm | 3172008A |
| P53 | DO-7 | 175 Lu | Cell Singnaling Technology  In-house conjugation | 48818 |

**Online Resource Table 2. CyTOF antibodies and metals used in panel 2.**

| **Antibody** | **Clone** | **Mass tag** | **Vendor** | **Cat no.** |
| --- | --- | --- | --- | --- |
| ***Surface panel 2:*** |  |  |  |  |
| CD45 | Hi30 | 89 Y | Fluidigm | 3089003B |
| CD66b | G10F5 | 141 Pr | BioLegend | 305102 |
| CD45RA | HI100 | 143 Nd | Fluidigm | 3143006B |
| CD38 | HIT2 | 144 Nd | Fluidigm | 3144014C |
| CD4 | RPA-T4 | 145 Nd | Fluidigm | 3145001B |
| CD8a | RPA-T8 | 146 Nd | Fluidigm | 3146001B |
| CD20 | 2H7 | 147 Nd | Fluidigm | 3147001B |
| CD16 | 3G8 | 148 Nd | Fluidigm | 3148004B |
| CD86 | IT2.2 | 150 Nd | Fluidigm | 3150020C |
| CD123 (IL-3R) | 6H6 | 151 Eu | Fluidigm | 3151001B |
| TIM3 | F38-2E2 | 154 Sm | Fluidigm | 3154010B |
| PD-1 | EH12.2H7 | 155 Gd | Fluidigm | 3155009B |
| CD11c | Bu15 | 159Tb | Fluidigm | 3159001B |
| CD14 | M5E2 | 160 Gd | Fluidigm | 3160001B |
| CTLA-4 | 14D3 | 161 Dy | Fluidigm | 3161004B |
| CD33 | WM53 | 163 Dy | Fluidigm | 3163023B |
| CD141 | M80 | 164 Dy | BioLegend  In-house conjugation | 344102 |
| CD45RO | UCHL1 | 165 Ho | Fluidigm |  |
| CD34 | 581 | 168 Er | BioLegend  In-house conjugation | 343531 |
| CD19 | HIB19 | 169 Tm | Fluidigm | 3169011B |
| CD3 | UCHT1 | 170 Er | Fluidigm | 3170001B |
| CD117 | 104D2 | 171 Yb | BioLegend  In-house conjugation | 313223 |
| CD56 | HCD54 | 173Yb | BioLegend  In-house conjugation | 318345 |
| HLA-DR | L243 | 174 Yb | Fluidigm | 3174001C |
| PD-L1 | 29E.2A3 | 175 Lu | Fluidigm | 3175017B |
| CD7 | CD7-6B7 | 176 Yb | BioLegend  In-house conjugation | 343111 |
| CD11b (Mac-1) | Mac-1 | 209 Bi | Fluidigm | 3209003B |
|  |  |  |  |  |
| ***Intracellular panel 2:*** |  |  |  |  |
| Caspase 3 (cleaved) | D3E9 | 142 Nd | Fluidigm | 3142004C |
| pP53 (S15) | 261366 | 149 Sm | R&D Systems  In-house conjugation | MAB18391 |
| pAkt (S473) | D9E | 152 Sm | Fluidigm | 3152005C |
| pSTAT1 (Y701) | 58D6 | 153 Eu | Fluidigm | 3153003C |
| p-p38 (T180/Y182) | D3F9 | 156 Gd | Fluidigm | 3156002C |
| pSTAT3 (Y705) | 4 | 158 Gd | Fluidigm | 3351607 |
| Ki67 | B56 | 162 Dy | Fluidigm | 3162012C |
| pNFkB (S529) | K10-895.12.50 | 166 Er | Fluidigm | 3166006A |
| Acetyl-P35 (K382) | REA529 | 167 Er | Miltenyi Biotec | 5170614144 |
| pS6 (S235/236) | N7-548 | 172 Yb | Fluidigm | 3172008A |

**Online Resource Table 3. Flow cytometry antibodies used in this study**

|  | **Antigen** | **Epitope** | **Clone** | **Conjugate** | **Manufacturer** |
| --- | --- | --- | --- | --- | --- |
| **Panel1** | NFkB | pS529 | K10-895.12.50 | PE | BD |
|  | STAT3 | pY705 | 4/P-STAT3 | Alexa Fluor 488 | BD |
|  | STAT5 | pY694 | 47 | Alexa Fluor 647 | BD |
| **Panel2** | STAT1 | pY701 | 4a | Alexa Fluor 488 | BD |
|  | p53 | pS37 | J159-641.79 | Alexa Fluor 647 | BD |
| **Panel3** | Akt | pT308 | J1-223.371 | PE | BD |
|  | p38 MAPK | pT180/pY182 | 36/p38 (pT180/pY182) | Alexa Fluor 488 | BD |
|  | ac-p53 | acK382 | L82-51 | Alexa Fluor 647 | BD |
| **Panel4** | p53 | aa 11-25 | DO-1 | PE | SC |
|  | ERK1/2 | pT202/pT204 | 20a | Alexa Fluor 488 | BD |
|  | STAT3 | pS727 | 49/p-Stat3 | Alexa Fluor 647 | BD |
| **Panel5** | CREB | pS133 | J151-21 | Alexa Fluor 488 | BD |
|  | anti-rpS6 | pS235/pS236 | N7-548 | Alexa Fluor 647 | BD |
| **Panel6** | p53 | pS15 | 16G8 | Alexa Fluor 488 | CST |
|  | STAT6 | pY641 | 18/P-Stat6 | Alexa Fluor 647 | BD |

SC; Santa Cruz Biotechnology, Santa Cruz, CA, USA. BD; BD Biosciences, San Diego, CA, USA. CST; Cell Signaling Technology, Danvers, MA, USA.

**Online Resource Table 4. Protein regulation relative to control after IFNα treatment**

|  | | | | **Fold change** | | |
| --- | --- | --- | --- | --- | --- | --- |
| **No.** | **Protein** | **Decyder number** | ***p-*value** | **IFNα-Le vs IFNα-2b*** | **IFNα-Le vs Ctr#** | **IFNα-2b vs Ctr#*** |
| **250 IU/mL IFNα-Le versus IFNα-2b** | | | | | | |
| **1** | **L-lactate dehydrogenase B chain (LDHB)** | 2331 | 0,00019 | 1,41 | -1,31 | 1,08 |
| **2** | **Aldose reductase (AKR1B1)** | 2292 | \| 0,00079 \| \| --- \| | \| 1,31 \| \| --- \| | -1,31 | -1,00 |
| **3** | **N-alpha-acetyltransferase 10 (NAA10)** | 2695 | \| 0,0039 \| \| --- \| | \| 1,32 \| \| --- \| | -1,46 | -1,11 |
| **4** | **Pyruvate kinase isozymes M1/M2 (PKM2)** | 1376 | \| 0,030 \| \| --- \| | \| 1,40 \| \| --- \| | -1,34 | 1,04 |
| **5** | **Catalase (CAT)** | 646 | \| 0,038 \| \| --- \| | \| 1,30 \| \| --- \| | -1,30 | -1,00 |
| **6** | **Pyruvate kinase isozymes M1/M2 (PKM2)** | 1381 | \| 0,040 \| \| --- \| | \| 1,39 \| \| --- \| | -1,43 | -1,03 |
| **7** | **26S protease regulatory subunit 7 (PSMC2)** | 1805 | \| 0,050 \| \| --- \| | \| -1,76 \| \| --- \| | -1,04 | -1,84 |
| **2000 IU/mL IFNα-Le versus IFNα-2b** | | | | | | |
| **1** | **14-3-3 protein epsilon (YWHAE)** | 2864 | \| 0,00014 \| \| --- \| | \| -1,47 \| \| --- \| | 1,53 | 1,09 |
| **2** | **28S ribosomal protein 23 (MRPS23)** | 3180 | \| 0,00032 \| \| --- \| | \| -1,30 \| \| --- \| | 1,12 | -1,16 |
| **3** | **40S ribosomal protein S4, X isoform (RPS4X)** | 2797 | \| 0,00093 \| \| --- \| | \| 1,36 \| \| --- \| | -1,25 | 1,09 |
| **4** | **Plastin-2 (LCP1)** | 1244 | \| 0,0015 \| \| --- \| | \| -1,48 \| \| --- \| | 1,16 | -1,27 |
| **5** | **Sorting nexin-5 (SNX5)** | 1628 | \| 0,0057 \| \| --- \| | \| -1,43 \| \| --- \| | 1,19 | -1,20 |
| **6** | **Acidic leucine-rich nuclear phosphoprotein 32 family member A (ANP32A)** | 2739 | \| 0,0076 \| \| --- \| | \| -1,56 \| \| --- \| | 1,36 | -1,15 |
| **7** | **Transgelin-2 (TAGLN2)** | 3189 | \| 0,0077 \| \| --- \| | \| -1,43 \| \| --- \| | 1,14 | -1,25 |
|  | **Heat shock protein HSP 90-beta (HSP90AB1)** | 829 | \| 0,0097 \| \| --- \| | \| -1,51 \| \| --- \| | 1,26 | -1,20 |
| **9** | **Alpha-enolase (ENO1)** | 1789 | \| 0,013 \| \| --- \| | \| -1,50 \| \| --- \| | 1,14 | -1,31 |
| **10** | **Protein DJ-1 (PARK7)** | 3207 | \| 0,015 \| \| --- \| | \| -1,39 \| \| --- \| | 1,15 | -1,20 |
| **11** | **14-3-3 protein epsilon (YWHAE)** | 2866 | \| 0,023 \| \| --- \| | \| -1,43 \| \| --- \| | 1,25 | -1,15 |
| **12** | **Spermidine synthase (SRM)** | 2651 | \| 0,034 \| \| --- \| | \| 1,33 \| \| --- \| | -1,09 | 1,22 |
| **13** | **Heat shock protein beta-1 (HSPB1)** | 3078 | \| 0,034 \| \| --- \| | \| -1,67 \| \| --- \| | 1,13 | -1,48 |
| **14** | **WD repeat-containing protein 1 (WDR1)** | 1104 | \| 0,035 \| \| --- \| | \| -1,31 \| \| --- \| | 1,15 | -1,14 |
| **15** | **78 kDa glucose-regulated protein (HSPA5)** | 3366 | \| 0,040 \| \| --- \| | \| -1,42 \| \| --- \| | 1,16 | -1,23 |
| **16** | **Heat shock cognate 71 kDa protein (HSPA8)** | 1158 | \| 0,041 \| \| --- \| | \| -1,41 \| \| --- \| | 1,17 | -1,20 |
| **17** | **T-complex protein 1 subunit alpha (TCP1)** | 1355 | 0,043 | -1,44 | 1,07 | -1,35 |
| **18** | **Annexin A5 (ANXA5)** | 2652 | 0,045 | -1,31 | 1,13 | -1,15 |

* Positive fold change indicates higher protein expression in IFNα-2b treated cells compared to IFNα-Le treatment; negative fold change indicates higher protein expression by IFNα-Le treatment compared to IFNα-2b treatment. # Positive fold change indicates higher protein expression by IFNα treatment; negative fold change indicates lower protein expression by IFNα treatment. DeCyder number refers to ID assigned by the DeCyder software; *p-*value was obtained by Students T-test in the DeCyder software.

**Online Resource Fig. 1. Gating strategy for CyTOF data**

After sample normalization, barcode deconvolution and manual doublet filtering based on DNA stain (ir191/193), unsupervised clustering was performed to identify cellular subsets using Phenograph. Phenograph was run on all samples from AML patients (n=48) and healthy donors (n=20), and all surface markers displayed in the figure were included as clustering channels. Phenograph identified a total of 19 metaclusters, which were manually classified as healthy PBMC populations or leukemic blasts (top panel). Leukemic blasts were defined as metaclusters not present in any of the healthy PBMC samples. Metaclusters defined as the same cell population was subsequently manually concatenated (middle panel), except MC3 and MC15 (both identified as DN T cells, but since MC15 had low expression of both CD4 and CD8 (likely background), these metaclusters were kept separate). The immunophenotype of the leukemic blasts in individual AML patients is shown as heatmap (lower panel).

**Online Resource Fig. 2. IFNα induces cell death in human MOLM-13.**

Human MOLM-13 cells were treated for 48 hours (n = 3) with 250 IU/mL or 2000 IU/mL recombinant IFNα-2b or IFNα-Le, 1 mM VPA or the combination of 1 mM VPA and 2000 IU/mL IFNα-Le, and investigated for abnormal nuclei, defined as apoptotic and necrotic cells, using the DNA stain Hoechst 33342. Error bars show standard error of mean. **A)** MOLM-13 cells showed statistically significant increased cell death when treated with IFNα or VPA. (Student’s unpaired, two tailed T-test; IFNα-Le 250 IU/mL versus 2000 IU/mL (*p =* 0.02), 2000 IU/mL IFNα-Le versus 2000 IU/mL IFNα-Le + 1 mM VPA (*p <* 0.0001) and 1 mM VPA versus 2000 IU/mL IFNα-Le + 1 mM VPA (*p =* 0.0014)). **B)** The combination of 1 mM VPA with 2000 IU/mL IFNα-Le showed synergism compared to single treatments (two-way ANOVA, * *p <* 0.01).

**Online Resource Fig. 3. VPA induce cell death in rat IPC-81 cells.**

**A)** Viability of rat IPC-81 cells (n = 3) was investigated by Annexin-V/propidium iodide (PI) after 48 hours treatment with recombinant IFNα-2b, IFNα-Le and/or 1 mM VPA. Cell death percentages were normalized to control cells. 1 mM VPA gave significantly increased cell death in IPC-81 cells compared to control and IFNα treated cells whereas IFNα did not increase the cell death effect of VPA (Student’s unpaired, two tailed T-test). **B)** IPC-81 cells were treated with IFNα-2b and IFNα-Le for 15 minutes and 48 hours, and investigated by phospho-flow cytometry. Only the highest dose of IFNα-Le (2000 IU/mL) induced phosphorylation of STAT1 at Y701 at both timepoints. Fold change is shown as log_2_.

**Online Resource Fig. 4. Phospho-signaling induced by recombinant IFNα-2b and human IFNα-Le in human AML MOLM-13 cells.**

Histograms of all investigated protein expression levels by IFNα mono-treatments after **A)** 15 minutes and **B)** 48 hours. Histograms of all investigated protein expression levels by 2000 IU/mL IFNα-Le alone or in combination with 1 mM VPA for **C)** 15 minutes and **D)** 48 hours. Fold change is shown as log_2_.

**Online Resource Fig. 5. STRING analysis of VPA and IFNα regulated proteins.**

Proteins with interactions found by database curating (turquoise lines) or determined experimentally (pink lines) are displayed. Pink circles indicate direct interactions between proteins regulated by the two drugs, and the green square indicates the focused group of proteins from known IFNα-induced pathways. YWHAE was found to connect to ERK1/2 (MAPK3/MAPK1) and AKT1, in addition to p53.

**Online Resource Fig. 6. Comparison of normal and complex karyotype AML patients for all markers evaluated by CyTOF**

PBMCs from AML patients treated with IFNα-2b and VPA and combination IFNα-2b/VPA for 48 h *ex vivo* were evaluated by CyTOF to investigate intracellular signaling and immune activation markers in defined cell subsets. Data are presented as arcsinh ratio relative to control. Statistics is based on difference between normal and complex karyotype. Kruskal-Wallis H test * *p* ≤ 0.05, ** *p* ≤ 0.01.

**Online Resource Fig. 7. Comparison of wild type and mutated NPM1 AML patients for all markers evaluated by CyTOF**

PBMCs from AML patients treated with IFNα-2b and VPA and combination IFNα-2b/VPA for 48 h *ex vivo* were evaluated by CyTOF to investigate intracellular signaling and immune activation markers in defined cell subsets. Data are presented as arcsinh ratio relative to control. Statistics is based on difference between NPM1 wild type and mutated patients. Kruskal-Wallis H test * *p* ≤ 0.05, ** *p* ≤ 0.01.

**Online Resource Fig. 8. Comparison of healthy and AML patient-derived PBMCs for all markers evaluated by CyTOF**

PBMCs from AML patients and healthy donors treated with IFNα-2b and VPA and combination IFNα-2b/VPA for 48 h *ex vivo* were evaluated by CyTOF to investigate intracellular signaling and immune activation markers in defined cell subsets. Data are presented as arcsinh ratio relative to control. Statistics is based on difference between healthy and AML patient-derived PBMCs. Kruskal-Wallis H test * *p* ≤ 0.05, ** *p* ≤ 0.01.

**Online Resource Fig. 9. Comparison of wild type and FLT3 ITD AML patients for all markers evaluated by CyTOF**

PBMCs from AML patients treated with IFNα-2b and VPA and combination IFNα-2b/VPA for 48 h *ex vivo* were evaluated by CyTOF to investigate intracellular signaling and immune activation markers in defined cell subsets. Data are presented as arcsinh ratio relative to control. Statistics is based on difference between FLT3 wild type and ITD positive patients. Kruskal-Wallis H test * *p* ≤ 0.05, ** *p* ≤ 0.01.

**References**

Alban, A., S. O. David, L. Bjorkesten, C. Andersson, E. Sloge, S. Lewis and I. Currie (2003). "A novel experimental design for comparative two-dimensional gel analysis: two-dimensional difference gel electrophoresis incorporating a pooled internal standard." Proteomics **3**(1): 36-44.

Bull, V. H., E. M. Fargestad, M. Strozynski and B. Thiede (2010). "Temporal proteome profiling of taxol-induced mitotic arrest and apoptosis." Electrophoresis **31**(11): 1873-1885.

Forthun, R., T. Sengupta, H. Skjeldam, J. Lindvall, E. McCormack, B. Gjertsen and H. Nilsen (2012). "Cross-species functional genomic analysis identifies resistance genes of the histone deacetylase inhibitor valproic acid." PloS one **7**(11).

Lacaze, N., G. Gombaud-Saintonge and M. Lanotte (1983). "Conditions controlling long-term proliferation of Brown Norway rat promyelocytic leukemia in vitro: primary growth stimulation by microenvironment and establishment of an autonomous Brown Norway 'leukemic stem cell line'." Leuk Res. **7**(2): 145-154.

Nair, A. B. and S. Jacob (2016). "A simple practice guide for dose conversion between animals and human." J Basic Clin Pharm **7**(2): 27-31.
